# Hydrazones and Thiosemicarbazones Targeting Protein-Protein-Interactions of SARS-CoV-2 Papain-like Protease

**DOI:** 10.3389/fchem.2022.832431

**Published:** 2022-04-11

**Authors:** Wiebke Ewert, Sebastian Günther, Francesca Miglioli, Sven Falke, Patrick Y. A. Reinke, Stephan Niebling, Christian Günther, Huijong Han, Vasundara Srinivasan, Hévila Brognaro, Julia Lieske, Kristina Lorenzen, Maria M. Garcia-Alai, Christian Betzel, Mauro Carcelli, Winfried Hinrichs, Dominga Rogolino, Alke Meents

**Affiliations:** ^1^ Center for Free-Electron Laser Science CFEL, Deutsches Elektronen-Synchrotron DESY, Hamburg, Germany; ^2^ Department of Chemistry, Life Sciences and Environmental Sustainability, University of Parma, Parma, Italy; ^3^ European Molecular Biology Laboratory Hamburg, DESY, Hamburg, Germany; ^4^ European XFEL GmbH, Schenefeld, Germany; ^5^ Institute of Biochemistry and Molecular Biology, Laboratory for Structural Biology of Infection and Inflammation, Department of Chemistry, University Hamburg, Hamburg, Germany; ^6^ Institute of Biochemistry, University Greifswald, Greifswald, Germany

**Keywords:** drug discovery, COVID-19, papain-like protease, x-ray crystallography, deubiquitination, SARS-CoV-2, lead compounds

## Abstract

The papain-like protease (PLpro) of SARS-CoV-2 is essential for viral propagation and, additionally, dysregulation of the host innate immune system. Using a library of 40 potential metal-chelating compounds we performed an X-ray crystallographic screening against PLpro. As outcome we identified six compounds binding to the target protein. Here we describe the interaction of one hydrazone (H1) and five thiosemicarbazone (T1-T5) compounds with the two distinct natural substrate binding sites of PLpro for ubiquitin and ISG15. H1 binds to a polar groove at the S1 binding site by forming several hydrogen bonds with PLpro. T1-T5 bind into a deep pocket close to the polyubiquitin and ISG15 binding site S2. Their interactions are mainly mediated by multiple hydrogen bonds and further hydrophobic interactions. In particular compound H1 interferes with natural substrate binding by sterical hindrance and induces conformational changes in protein residues involved in substrate binding, while compounds T1-T5 could have a more indirect effect. Fluorescence based enzyme activity assay and complementary thermal stability analysis reveal only weak inhibition properties in the high micromolar range thereby indicating the need for compound optimization. Nevertheless, the unique binding properties involving strong hydrogen bonding and the various options for structural optimization make the compounds ideal lead structures. In combination with the inexpensive and undemanding synthesis, the reported hydrazone and thiosemicarbazones represent an attractive scaffold for further structure-based development of novel PLpro inhibitors by interrupting protein-protein interactions at the S1 and S2 site.

## Introduction

Within the last 20 years, the world has been confronted with three emerging zoonotic coronaviruses, namely severe acute respiratory syndrome coronavirus (SARS-CoV-1), middle east respiratory syndrome coronavirus (MERS-CoV) and SARS-CoV-2, which collectively have claimed more than five million victims so far (de Wit et al., 2016; WHO, 2021). Previous research on coronaviruses together with recent advances in biotechnology enabled the rapid development of novel vaccines in the current COVID-19 pandemic caused by SARS-CoV-2 (V’kovski et al., 2020; Tregoning et al., 2021). Although current vaccines offer good protection against most virus variants, there is still an urgent demand for complementary antiviral drugs that are suitable for patients who are already infected, cannot be vaccinated, are immune compromised or do not have access to any vaccination. The occurrence of immune escape variants further highlights the need for alternative treatments.

The high similarity to SARS-CoV-1 in genome sequence and viral replication helped to rapidly understand the biology of the newly emerged coronavirus SARS-CoV-2 (Lu et al., 2020; Zhou et al., 2020). Both genomes encode 16 non-structural proteins (nsps) including two cysteine proteases, which are essential for viral replication. These proteases are named main protease (Mpro, alternatively 3C-like protease) and papain-like protease (PLpro) and are responsible for the sequential proteolytic cleavage of the two polyproteins 1a and 1ab, which are the primary translation products of the viral genome (Chan et al., 2020). While Mpro releases 11 nsps from the polyprotein chains including itself (Zhang et al., 2020), PLpro is a component of the largest multidomain replicase subunit (nsp3) and recognizes the sequence LXGG (residues P4-P1) in between nsps 1–4 (Barretto et al., 2006). Both proteases, but in particular Mpro, have been the target of several extensive drug development projects (Citarella et al., 2021; Günther et al., 2021). As druggable target, PLpro has the advantage that its catalytic activity is not only essential for viral propagation but also interferes with the host innate immune system (Vabret et al., 2020). Post-translational modifications (PTM) like the conjugation with ubiquitin and ubiquitin-like proteins, including interferon-stimulating gene 15 (ISG15), regulate the cellular location of proteins, their stability and, by this, their antiviral effect (Mevissen and Komander, 2017). PLpro can revoke these PTMs by hydrolysing the isopeptide bond at the C-terminus of cellular ubiquitin (Ub) and ISG15, which results in a dysregulation in the production of cytokines and chemokines and type I interferon response (Liu et al., 2021; Munnur et al., 2021). Together with other dysregulations this leads to an excessive immune response (“cytokine storm”) that causes additional collateral damage and is widely responsible for the substantial morbidity and mortality in COVID-19 patients. Targeting PLpro with newly designed drugs can therefore not only inhibit the viral replication but presumably also promote the host immune function, rendering PLpro as a highly attractive and prioritised drug target.

**FIGURE 1:**
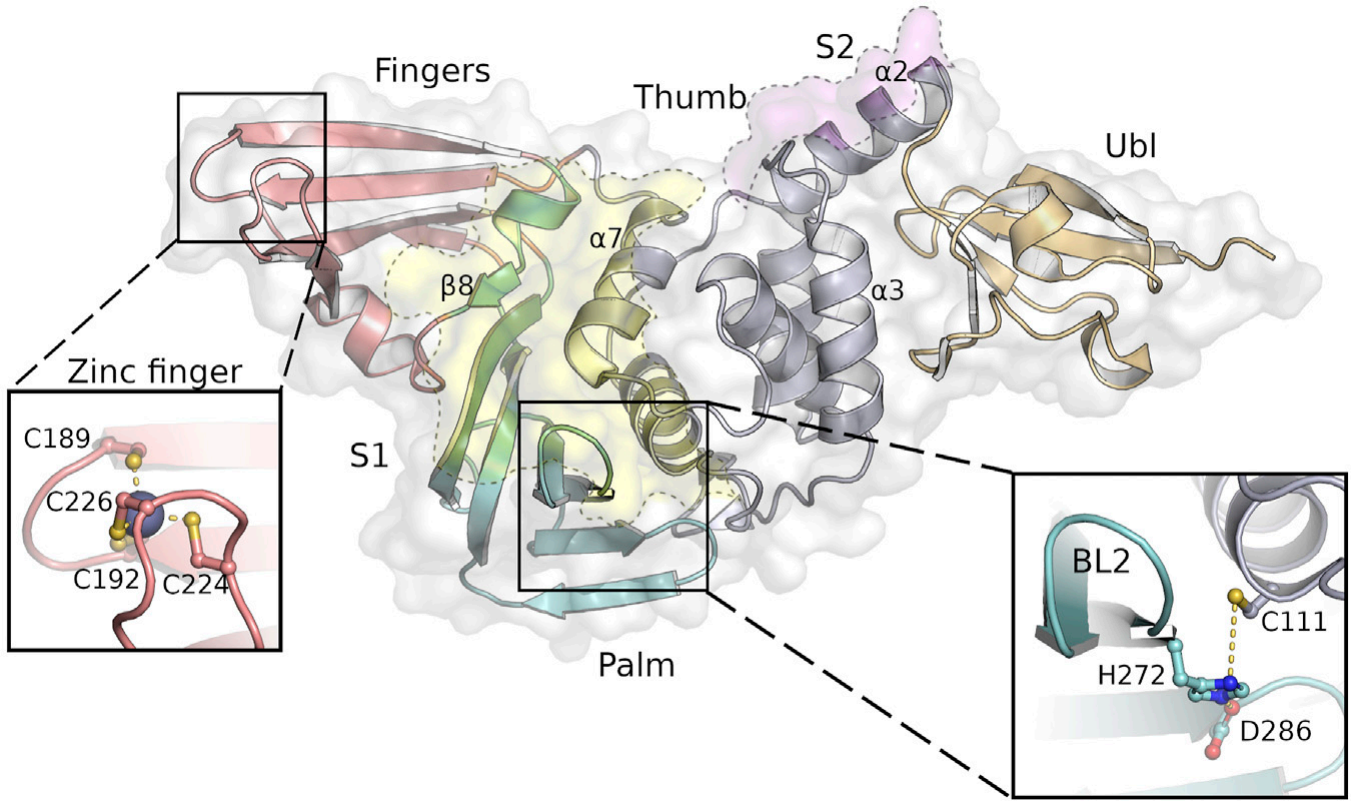
SARS-CoV-2 PLpro structure with the four domains and important features of this protein indicated as follows: fingers (salmon), palm (cyan), thumb (purple) and Ubl domain (orange). The substrate binding sites S1 and S2 are highlighted as yellow and pink areas, while the close-ups show the tetrahedral coordinated zinc-ion at the finger tips and the highly conserved catalytic triad next to the flexible BL2. Secondary structure motifs further discussed are labeled accordingly.

**FIGURE 2:**
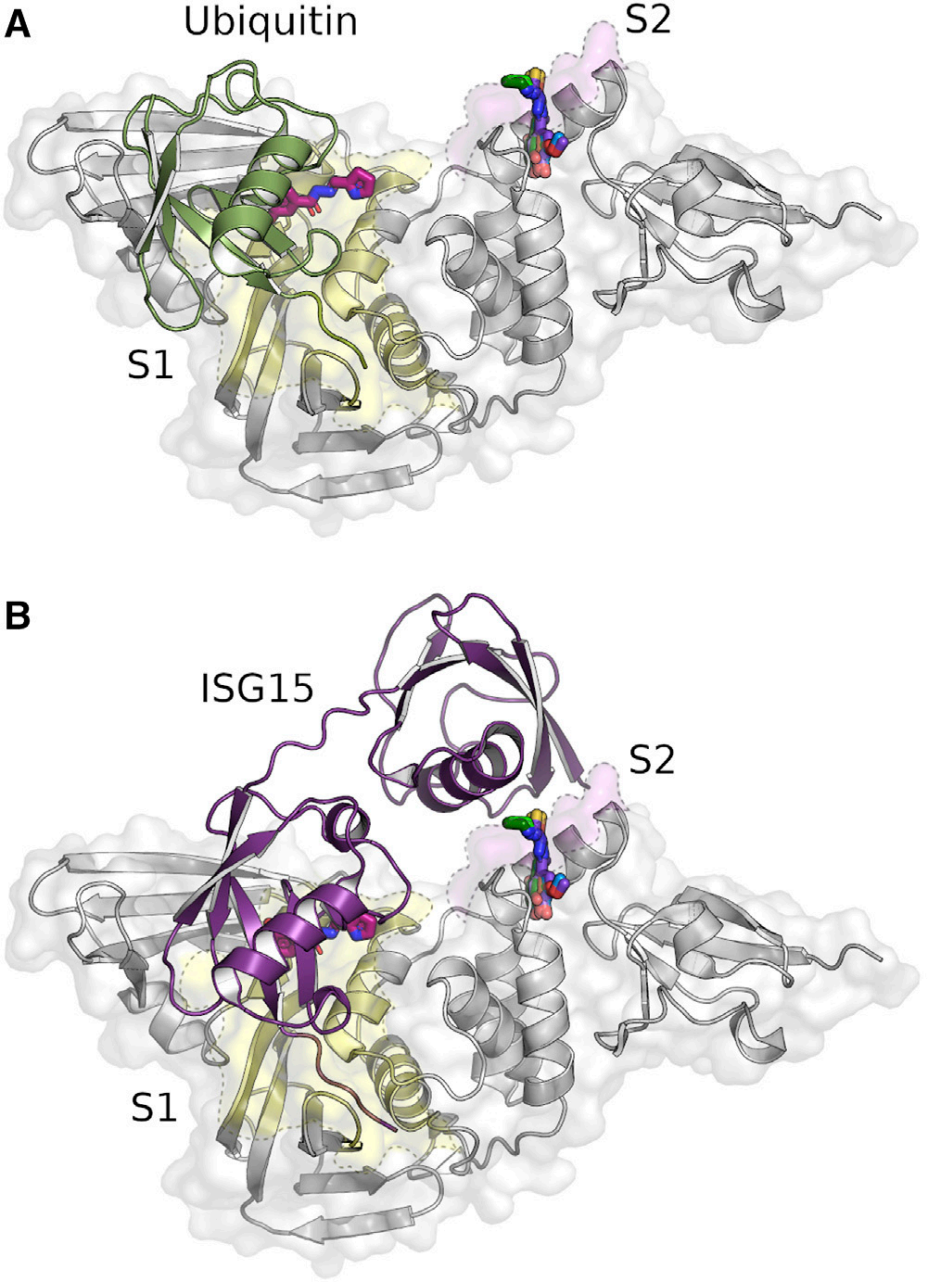
Identified compounds bind near the S1 (yellow) and S2 site (pink) of PLpro. Crystal structure of SARS-CoV-2 PLpro in complex with the identified hydrazone H1 and thiosemicarbazone binders T1-T5 overlayed with the binding ubiquitin [**(A)**, green, PDB: 6xaa] and ISG15 molecules [**(B)**, purple, PDB: 7rbs]. Five of the six identified ligands (T1–T5) are in a deep pocket near the S2 site, while the hydrazone H1 (magenta) is located at the end of the S1 site.

PLpro is a monomer in solution and has a right-handed ubiquitin specific protease (USP) fold which consists of four domains—the N-terminal ubiquitin-like (Ubl) domain, the thumb, palm and fingers domain (Ratia et al., 2006) (Figure 1). At the tip of the fingers a zinc ion is coordinated, which is essential for protease activity (Barretto et al., 2005). The peptide bond cleavage in the active site is catalyzed by a conserved catalytic triad (C111-H272-D286) that is located at the interface of the thumb and palm domain. Identification of specific active site inhibitors for PLpro, including approaches to analyse peptidic, non-peptidic and “dual target” inhibitors (Rut et al., 2020; Zmudzinski et al., 2020; Shen et al., 2021), is particularly challenging due to a rather “featureless” active site and a high similarity to host deubiquitinases compared to proteases like Mpro. Access to the PLpro active site is regulated by a flexible blocking loop 2 (BL2) which is involved in substrate binding (Báez-Santos et al., 2015). PLpro binds ubiquitin and ubiquitin-like proteins at two distinct sites, S1 and S2, thereby providing specificity for K48-polyubiquitin (K48-Ub_2_) and ISG15 (Figures 1, 2) (Békés et al., 2016; Klemm et al., 2020). These sites do not refer to the commonly used notation of peptide substrate-binding sites of proteases according to Schechter and Berger (Schechter and Berger, 1967).

While current research focuses primarily on inhibitors that bind to the S1 site and interfere with the deubiquitinase activity of PLpro, the aim of our work was to find inhibitors, as for example disulfiram (Sargsyan et al., 2020), that interact with the ion in the zinc finger but not with the active site of the protease. Although the zinc finger and the catalytic site are about 40 Å apart, the correct zinc coordination is mandatory for structural stability and protease activity of SARS-CoV-2 PLpro (Barretto et al., 2005). Inhibition of a viral enzyme by coordinating one or more metal cofactors represents a successful strategy in the development of novel therapeutic agents (Chen et al., 2019); in particular, chelation of Zinc (II) ions by N-acylhydrazones seems related to interesting biological effects (Hsu et al., 2012; Huan et al., 2020). Some data indicating that this approach is applicable to SARS-CoV-2 viral proteins have already been disclosed (te Velthuis et al., 2010; Panchariya et al., 2021). Thus, we defined a small in-house library of 40 previously synthesized quinolone, hydroxyquinoline, thiosemicarbazone and hydrazone compounds (Supplementary Table S1), that have been proved to be protein inhibitors in other relevant viral metalloenzymes (Rogolino et al., 2015; Carcelli et al., 2016; Carcelli et al., 2017) and investigated their interaction with PLpro by high-resolution X-ray crystallography and additional *in vitro* and *in silico* analyses.

## Materials and Methods

### Cloning, Expression and Purification

The PLpro polypeptide corresponding to amino acid residues 746–1,060 of SARS-CoV-2 nsp3 (YP_009742610.1) was cloned into pETM11 with an additional N-terminal His6-tag and TEV-cleavage site. The construct was overexpressed in *E. coli* Rosetta (DE3) according to a previously published protocol (Studier, 2005) and purified for subsequent crystallization. Lysis was carried out in 50 mM NaH_2_PO_4_ buffer supplemented with 150 mM NaCl and 10 mM imidazole at pH 7.2 using ultrasound for cell disruption. After separation of cell fragments and dissolved protein, a subsequent Ni-NTA chromatography step was used to extract the fusion protein. The cleavage of the histidine tag was achieved by TEV protease during an overnight dialysis step at 8°C. After removing the TEV protease and His_6_-tag *via* Ni-NTA resin, a final size exclusion chromatography was performed using a HiLoad 16/600 Superdex 75 column attached to an ÄKTA purifier (GE Healthcare) to purify the protein to homogeneity in 50 mM Tris-HCl, 150 mM NaCl and 1 mM TCEP at pH 7.8.

### Protein Crystallization

Crystallization of PLpro was achieved by mixing 0.2 μL protein (20 mg/ml) with 0.1 μL of reservoir solution consisting of 100 mM Tris-HCl buffer pH 8.0, 10% (w/w) glycerol and 0.8 M NaH_2_PO_4_/1.2 M K_2_HPO_4_. The crystallization drops were prepared using an Oryx6 pipetting robot (Douglas Instruments) and equilibrated by sitting drop vapor diffusion against 40 μL reservoir solution. Bipyramidal crystals appeared within a few days at 4°C and reached a final size of approximately 100 μm. Crystals were soaked with reservoir solution containing up to 5 mM of the respective compound with a final DMSO concentration of 5%. After 24 h the soaked crystals were harvested and cryo-cooled in liquid nitrogen for subsequent X-ray diffraction data collection.

### Data Collection, Processing, Hit Finding and Refinement

Data collection was performed at beamline P11 at the PETRA III storage ring at DESY in Hamburg (Germany). The obtained data sets were processed with DIALS (Winter et al., 2018). The results for each data set were subjected to automated structure refinement using phenix (Liebschner et al., 2019) followed by pan data set density analysis (PanDDA) (Pearce et al., 2017) using default parameters. The results were manually inspected for hits. Identified hits were further refined by alternating rounds of refinement using phenix.refine (Afonine et al., 2018) and manual model building in COOT (Emsley and Cowtan, 2004). Diffraction data quality indicators and refinement statistics for all data sets are summarized in Supplementary Table S2.

### Fluorescence Polarization-Based Activity Assay

Assays were performed using Ub-KG-TAMRA (UbiQ-012, UbiQ bio) and human ISG15-KG-TAMRA (UbiQ-287, UbiQ bio) to determine the inhibitory potential of the selected compounds on PLpro activity following the protocol described by Klemm et al. (2020). With substrate concentration kept at 150 nM, PLpro concentration was set to 500 nM for Ub-TAMRA- and to 5 nM for ISG15-TAMRA-cleavage. The protein was preincubated with 500 µM or 5 µM of the selected compounds for 20 min at RT before addition of substrate. Reactions were monitored using a Spark 20 M plate reader (Tecan) with optical settings for the TAMRA fluorophore (excitation: 540 nm, emission: 590 nm). Data was plotted and analyzed using the software Origin (OriginLab).

### Nano Differential Scanning Fluorimetry

Nano Differential Scanning Fluorimetry (nDSF) measurements were performed with a Nanotemper Prometheus NT.48 fluorimeter (Nanotemper) controlled by PR. ThermControl using Prometheus Premium grade capillaries (Nanotemper). The excitation power was adjusted to obtain fluorescence counts above 2,000 RFU for 330 and 350 nm. For all measurements a PLpro concentration of around 2 mg/ml in 50 mM Tris-HCl, 150 mM NaCl, 1 mM TCEP, pH 7.8 containing 5% DMSO was used with varying ligand concentrations. For the initial melting temperature screening, we have used a ligand concentration of 500 µM (468 µM for T3). For the fluorescence titrations 1:1 dilution series with 15 points (19 points for T5) of ligands was created and then the protein solution was added. Ligand concentrations range from 500 µM to 28 nM (5 mM–19 nM for T5). After incubation of 30 min, the solutions were transferred to capillaries and transferred to the Prometheus fluorimeter for the measurement.

Data were analyzed and visualized with self-written python scripts using the Python modules Numpy (Oliphant, 2006; van der Walt et al., 2011), Matplotlib (Hunter, 2007), Scipy (Virtanen et al., 2020) and Pandas (McKinney, 2010) and the publicly available eSPC data analysis platform (Burastero et al., 2021). The fluorescence titration of T5 was fitted with a simple 1:1 binding model.

$$F_{350\text{nm}}\left({\left[L\right]}_{0}\right)=F_{upper}+\left(F_{upper}-\;F_{lower}\right)*\left(1-\alpha \left({\left[L\right]}_{0}\right)\right)$$ (1)

$$\alpha \left({\left[L\right]}_{0}\right)=\left({\left[P\right]}_{0}-K_{D}-{\left[L\right]}_{0}+\sqrt{{\left({\left[P\right]}_{0}+{\left[L\right]}_{0}+K_{D}\right)}^{2}-4*{\left[P\right]}_{0}*{\left[L\right]}_{0}}\right)/\left(2*{\left[P\right]}_{0}\right)$$ (2)

### Molecular Docking

Molecular docking was performed using AutoDock4.2.6 (Morris et al., 2009). Protein coordinates were obtained from the corresponding PDB-files (7qcg, 7qch, 7qci, 7qcj, 7qck, and 7qcm) and processed with AutoDockTools. The covalently connected ligand structures were chosen depending on the structural overlay visible when aligning the corresponding PLpro structures with PDB-files 7ofs, 7oft or 7ofu (Supplementary Table S3) and prepared using eLBOW (Moriarty et al., 2009) and AutoDockTools. Grid maps with a box of 45 × 35 × 35 grid points (T1-T5) or with a box of 45 × 35 × 45 grid points (H1) with 0.375 Å spacing were set around the corresponding binding sites. The docking calculations were performed using the Lamarckian genetic algorithm (GA) combining a global search with a local search (Morris et al., 1998). The most favorable structure with the highest binding energy in the maximum cluster of the docked conformations was chosen as the representative structure in Autodock. To calculate reliable binding energies the representatives structures were further processed with Haddock (van Zundert et al., 2016; Honorato et al., 2021) and Prodigy webserver (Kurkcuoglu et al., 2018; Vangone et al., 2019).

### Synthesis of Compounds

Compounds 1–40 were synthesized following literature methods according to references reported in Supplementary Table S1. Characterization of compounds T1–T5 and H1 is reported in the Supplementary Information Paragraph.

## Results

### Compounds Bind at Two Different Substrate Binding Sites in SARS-CoV-2 PLpro

In total 71 diffraction data sets from crystals with 40 different compounds were collected with high resolution limits ranging from 1.6—3.0 Å. In the subsequent analysis of the X-ray diffraction data nine compounds were identified binding to PLpro in PanDDA difference electron density maps (Pearce et al., 2017). Out of these nine hits the binding modes of six different compounds could be unambiguously determined in data sets with a diffraction limit of 1.75–1.92 Å (Figure 2).

**FIGURE 3:**
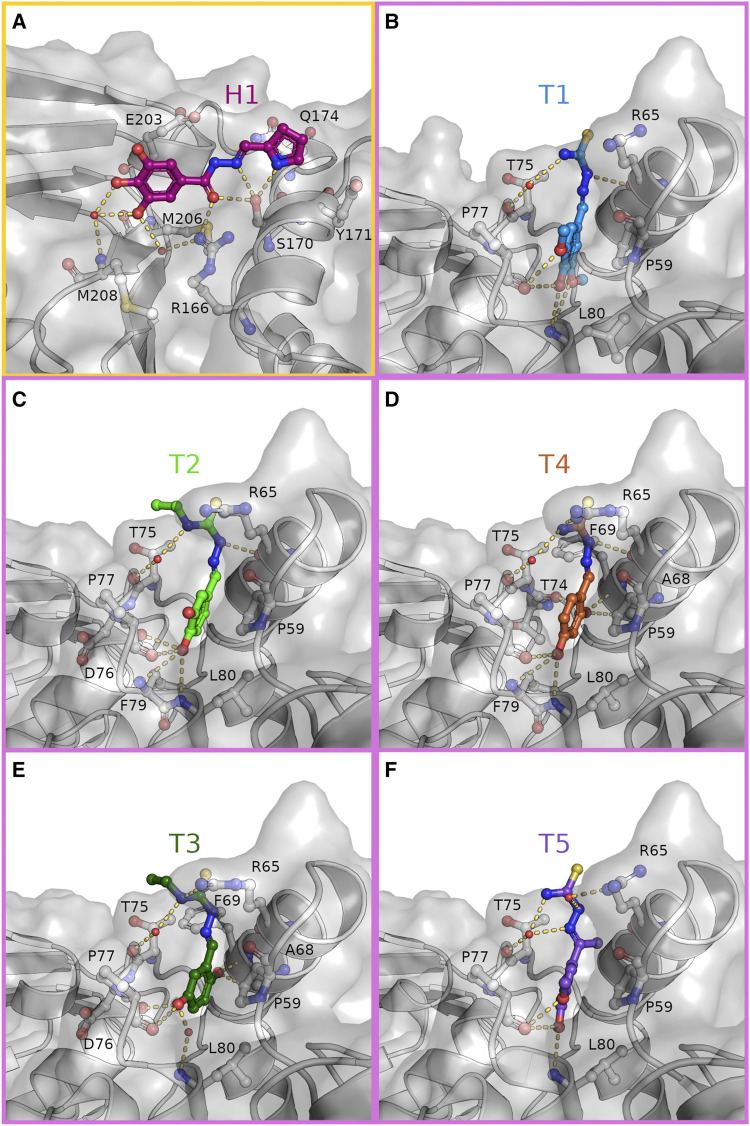
Hydrazone and thiosemicarbazones are stabilized in their binding positions by an extensive hydrogen bonding network. Close-up view on the binding of H1 [**(A)**, magenta] to the S1 site (yellow frame) and T1-T5 **(B–F)** to the S2 site (pink frames). Compounds and interacting residues are shown as sticks with compounds highlighted by individual colouring. Possible interactions within hydrogen bonding distance with the surrounding residues are shown as dashes.

Interestingly, none of the anticipated zinc binders was found near the zinc binding site but instead at a previously undescribed groove within the S1 site (Figure 2A) and a pocket between the S2 binding site and Ubl domain (Figure 2B). The S1 site is targeted by hydrazone H1, whereas five thiosemicarbazones (T1-T5) bind to the S2 site (Supplementary Table S1, highlighted ligands). For both binding sites, one compound each showed superior electron density maps, where the compound could be refined with full occupancy (Supplementary Figure S1). In all six structures compound binding induced only local rearrangements at the binding sites with an overall r.m.s.d. of 0.15–0.25 Å compared to the ligand-free structure (PDB: 7nfv). All compounds bind non-covalently, primarily through hydrogen bonds and π-alkyl interactions (Figure 3).

The hydrazone compound H1 binds in a polar groove at the S1 site of PLpro between β-strand β8 (M206—M208) of the palm and helix α7 (V165—H175) of the thumb domain. In this groove H1 is stabilized by eight hydrogen bonds (Figure 3A and Supplementary Figure S2A). Here, the hydroxyl side chain of S170 acts as a hydrogen bond acceptor and donor to the nitrogen atoms of the pyrrole and imino moiety, respectively. The central carbonyl oxygen of H1 is the hydrogen bond acceptor for side chain of R166 and again S170, while two phenolic hydroxyl groups of the terminal benzene substituent are hydrogen bonded *via* one water molecule to the amide nitrogen of M208. One of these hydroxyl groups forms a second hydrogen bond to the main chain carbonyl of M206, whereas the third hydroxyl is solvent exposed. When compared with the ligand-free structure (PDB: 7nfv) S170 is observed in an alternative rotameric state, which is moved 1.7 Å by the attractive interaction towards H1 (Supplementary Figure S3A). An additional side chain rearrangement in the surrounding residues is observed for residue Q174, which adopts two side chain conformations in the ligand-free structure but prefers only one conformation in the H1 bound structure. In this position the carboxamide side chain has moved by 3.9 Å (conformation A) and 0.9 Å (conformation B), respectively, to complement the compound binding by a hydrogen bond to the *π*-system of the hydrazone. Further stabilization is achieved by C-H···*π* interactions between the phenyl ring of the compound with the side chain of E203 and the pyrrole ring with the aromatic side chain of Y171.

The thiosemicarbazone derivatives T1–T5 all bind in a deep pocket close to the S2 site with a volume of about 70 Å^3^ (Figures 3B–F and Supplementary Figures S2B–F). This cavity is enclosed by helices α2 (D62—Y72) and α3 (residues P77—K92) of the thumb domain and loop 7 connecting the Ubl with the thumb domain. Here the phenolic system of the compounds points into the N-terminal turn (residues P77 –L80) of the thumb helix α3 suitable to interact with the helical dipole. In this position the aromatic plane becomes a part of the hydrophobic interface between T75, P77 and the adjacent Ubl domain residue P59 through C-H···*π* interactions (Figures 2, 3B). The substitutions on the phenolic system in T1–T5 form a hydrogen bonding pattern exclusive with main chain atoms of the N-terminal helical turn of α3. The different arrangements of hydroxyl- and methoxy substituents at the benzene ring determine the final orientations of the phenolic system, displacing the ring system in plane. While the overall position of the thiosemicarbazide moiety of T1–T5 are almost identical with polar interactions to the side chain and main chain carbonyl of R65 (α2), minor differences are observed due to the variation of the hydroxylation pattern. The specific binding modes for each compound are explained in more detail in the following.

Within the group of thiosemicarbazones, compound T1 showed the best difference electron density map and was refined with full occupancy. Compounds T2–T5 could only be refined with lower occupancy, but highly resemble the T1 binding position (Supplementary Figure S1). The more defined electron density of T1 is probably caused by the advantageous pattern of hydroxyl and methoxy substituents at the benzene ring (Figure 3B). Especially the *para*-hydroxyl group, present in four of the thiosemicarbazones, plays a key role in anchoring the molecule at the bottom of the binding pocket, as this substitution acts as a hydrogen bond donor and acceptor to the backbone carbonyl of P77 and the amide N-H of L80, respectively. Only in T3 this interaction is guided by a phenolic hydroxyl group in *meta*-position (Figure 3E). Due to the attractive hydrogen bonding pattern to the N-terminal turn of helix α3 the plane of the benzene ring of T3 is shifted to place its *meta*-hydroxyl substituent similar to the *para*-hydroxyl groups of T1, T2, T4, and T5. While the direct hydrogen bond to L80 is substituted by the main chain carbonyl of D76, T3 still interacts with L80 through a water mediated hydrogen bond.

For all five thiosemicarbazones, the thiosemicarbazide fragment is sandwiched by side chains R65 (α2) and T75 (loop α2, α3) and its terminal thiourea points towards the solvent. This fragment forms polar interactions with the side chain of R65. In case of T1, T2 and T4 this moiety is held in position by a hydrogen bond of the hydrazine N-H to the backbone carbonyl of residues R65 (Figures 3B–D).

Thiosemicarbazones T2 and T3 both feature an ethyl chain at N3 of the thiourea moiety, which extends in the direction of T75 and thereby reduces the solvent accessible area of this residue (Figures 3C,E). T5 is the only compound with a methyl substituent at the C=N bond of the thiosemicarbazone. This methyl group points towards the backbone atoms of the C-terminal turn of α2 (F69) displacing the T5 benzene axis slightly, while the phenolic *para*-hydroxyl still determines the overall position inside the binding pocket (Figure 3F).

In comparison to the ligand-free structure again two conformational rearrangements in the surrounding residues are observable (Supplementary Figure S3B). As a result of the T1 binding the guanidyl group of R65 is displaced by 3.0 Å to open the binding pocket for the thiourea moiety of this compound. To fully accommodate this ligand further opening of the pocket is induced by the sterical requirements of the methoxy groups of T1, which enable the side chain movement of L80 by 1.7 Å compared to the ligand-free structure.

### Compound Binding Indicates Sterical Hindrance for Substrate Binding

**FIGURE 4:**
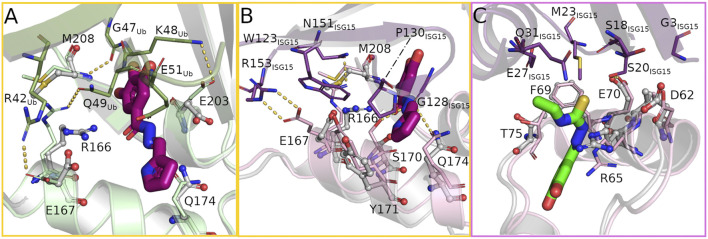
The bound compounds interfere with key residues for substrate binding at the S1 (yellow frame) and S2 (pink frame) site. **(A)** Overlay of ubiquitin bound to PLpro (PDB: 6xaa, PLpro light green, ubiquitin green) and the hydrazone bound structure (PLpro grey, H1 magenta) showing the rearrangement of several PLpro-ubiquitin interacting side chains to form hydrogen bonds with the compound. **(B,C)** Overlay of PLpro complexed with human ISG15 (PDB: 7rbs, PLpro light pink, ISG15 purple) and PLpro bound with hydrazone H1 and thiosemicarbazone T2 (PLpro grey, H1 magenta, T2 green) at the S1 and S2 sites respectively highlighting the structural differences in binding the compound or substrate.

All six compounds bind in close proximity to the S1 and S2 sites that are responsible for binding the native PLpro substrates ubiquitin and ISG15. Superposition of these PLpro complex structures (PDB: 6xaa, 7rbs, respectively) with our structures suggests that in particular compound H1 is partially overlapping with the natural substrate binding site. In addition, we observed altered interactions of key PLpro residues involved in substrate binding (Figure 4).

Ubiquitin binds to the S1 site of PLpro by sitting on the palm domain and is additionally held in position by the fingers domain (Figure 2A). In addition to numerous nonpolar interactions, multiple intermolecular hydrogen bonds within the active site and the adjacent S1 site support this binding. At the core of the ubiquitin binding interface around residue I44_Ub_ are four non-covalent bonds between ubiquitin and PLpro (one hydrogen bond, three salt bridges), all of which are likely disrupted by binding of H1 (Figure 4A). Interestingly only the hydrogen bond is lost due to direct sterical hindrance (M208/G47_Ub_), whereas the three salt bridges (R166/Q49_Ub_, E167/R42_Ub_, and E203/K48_Ub_) are disrupted due to side chain reorientations towards the bound ligand. While E203 alters its conformation without direct interaction with the ligand, residues R166 and E167 are not only attracted by H1 but even form alternative hydrogen bonds with each other and H1 to support a highly polar ligand environment. In addition to these changes the ligand further interferes sterically with residue E51_Ub_.

The C-terminal domain of human ISG15 binds mainly to the thumb domain at the S1 site of PLpro and interacts with a different set of residues compared to the PLpro/ubiquitin complex (Figure 2B). The key interaction sites mediating the contacts between PLpro and ISG15 can be found around ISG15 residues W123_ISG15_ and P130_ISG15_ (Fu et al., 2021; Osipiuk et al., 2021) (Figure 4B). Within PLpro an overall upward shift of 1.7 Å in the interacting helix α7 becomes visible that strengthens ISG15 binding. Five hydrogen bonds between G128_ISG15_ and S170/Q174, N151_ISG15_ and R166 and R153_ISG15_ and E167 thereby stabilize the interface. Especially the latter one contributes to the interaction of the proteins, as the side chain not only forms two hydrogen bonds but further has an aliphatic interaction with W123_ISG15_. Y171 further stabilizes ISG15 binding by π-stacking interactions with P130_ISG15_. Superposition of the H1 complex with the PLpro/ISG15 structure reveals a variety of side chain rearrangements which show that ISG15 binding could not only be affected by direct overlap with the compound but also by multiple lost interactions. While the hydrogen bonds with G128_ISG15_ are likely disrupted by the sterical clash of H1 and the ISG15 loop comprising residues F126_ISG15_ to P130_ISG15_, the interaction between N151_ISG15_ and R166 is impaired due to the movement of the arginine side chain by 4.0 Å that forms a hydrogen bond with H1 in the complex structure. This rearrangement is accompanied by two additional side chain movements of residues E167 and M208. As a result, residues W123_ISG15_ and R166 show a significant overlap with these residues, which likely further destabilizes ISG15 binding at the S1 site.

The binding of the N-terminal domain of human ISG15 to the S2 site is mediated mainly by interactions between helix α2 of PLpro and two β-strands of ISG15 (Figure 4C). While residues G3_ISG15_, S20_ISG15_ and M23_ISG15_ form a hydrophobic patch that interacts with V66, additional stabilization is formed by a hydrogen bond between S20_ISG15_ and E70 and a C-H···*π* interaction between M23_ISG15_ and F69. For the ISG15 helix interacting with the PLpro loop containing T75 also residues E27_ISG15_ and Q31_ISG15_ contribute to the binding. As the thiosemicarbazone compounds bind in close proximity to the S2 site, but not prominently at the interface of the N-terminal domain of ISG15 and PLpro, the potential for direct sterical interference by these ligands is rather small compared to H1. However, a closer look at the surrounding residues suggests that the thiosemicarbazones may alter the polarity and flexibility of the S2 binding site. T1–T5 are located at the interface of the Ubl and thumb domain and could interfere with the mobility of the Ubl domain by disturbing the interaction network between residues P59, P77 and T75. T75 is highly relevant for ISG15 binding, as it can directly alter the conformation of F69 (Bosken et al., 2020). T2 and T3 may further affect the interaction of PLpro T75 with E27_ISG15_, as the ethyl N3 substituent is positioned close to T75 (Figure 4C).

**FIGURE 5:**
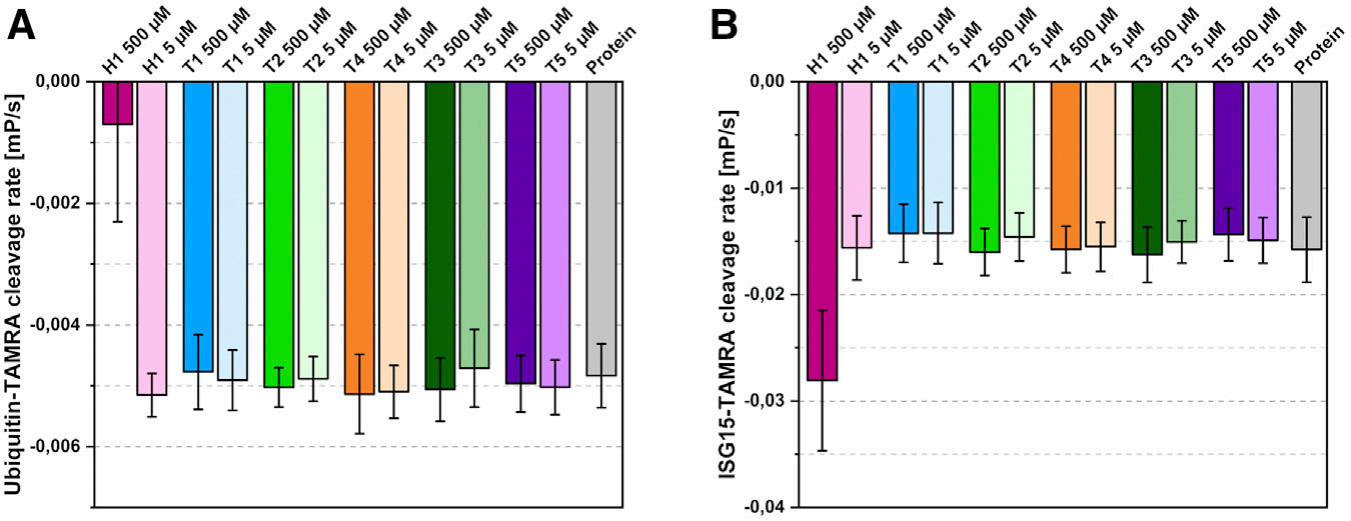
Effects of the identified binders on substrate cleavage rates. **(A)** Ubiquitin-cleavage rate of PLpro in the presence of 500 µM or 5 µM compound. **(B)** ISG15-cleavage rate of PLpro in the presence of 500 µM or 5 µM compound. Compounds coloured according to Figure 3; protein shown in grey. Average of four independent experiments with standard deviation shown.

To test the inhibitory potential of the six compounds on substrate turnover, T1-T5 and H1 were examined in a fluorescence polarization assay (Figure 5) using ubiquitin and human ISG15 as substrates. The results show the highly divergent turnover rates for both substrates and the preference of SARS-CoV-2 PLpro for ISG15 (Freitas et al., 2020). While the thiosemicarbazones show no inhibitory effect on substrate turnover at the tested concentrations, a significant inhibition of the ubiquitin cleavage can be detected for 500 µM of hydrazone H1. The fivefold decrease in substrate turnover is consistent with the expected sterical interference at the S1 site as mentioned above. Surprisingly, ISG15 turnover in the presence of 500 µM of H1 is not reduced but rather increased twofold compared to ligand-free PLpro. For the other compounds, no inhibitory effect was detected at the tested concentrations.

As missing inhibition can be caused by low binding affinities of the compounds, additional nano DSF measurements were performed (Supplementary Figure S4). The thermal shift assay showed a considerable stabilization for all compounds (Supplementary Figure S4A) in combination with a strong quenching of the intrinsic protein fluorescence caused by the ligands. This strong fluorescence quenching renders the thermal unfolding curve almost featureless for some of the ligands. The signal at 350 nm shows the clearest transitions and was therefore selected to calculate the melting temperature shifts. To estimate the binding affinity of the compounds we performed nDSF/fluorescence titrations, which are shown in Supplementary Figures S4B, S4C. These titrations indicate dissociation constants in the high micromolar range for all tested compounds. However, due to the low solubility of the ligands, high enough concentrations for a reliable K_D_ determination by isothermal analysis (Bai et al., 2019; Niebling et al., 2021) or the initial fluorescence fit are missing. One exception is T5, for which higher concentration data are available. A fit of the initial fluorescence at 330 nm yields an apparent K_D_ of approximately 200 µM. The fluorescence titration of T5 is very similar to the other tested ligands, therefore we expect dissociation constants in the same range.

### Docking Studies Reveal Lead Compound Potential

**FIGURE 6:**
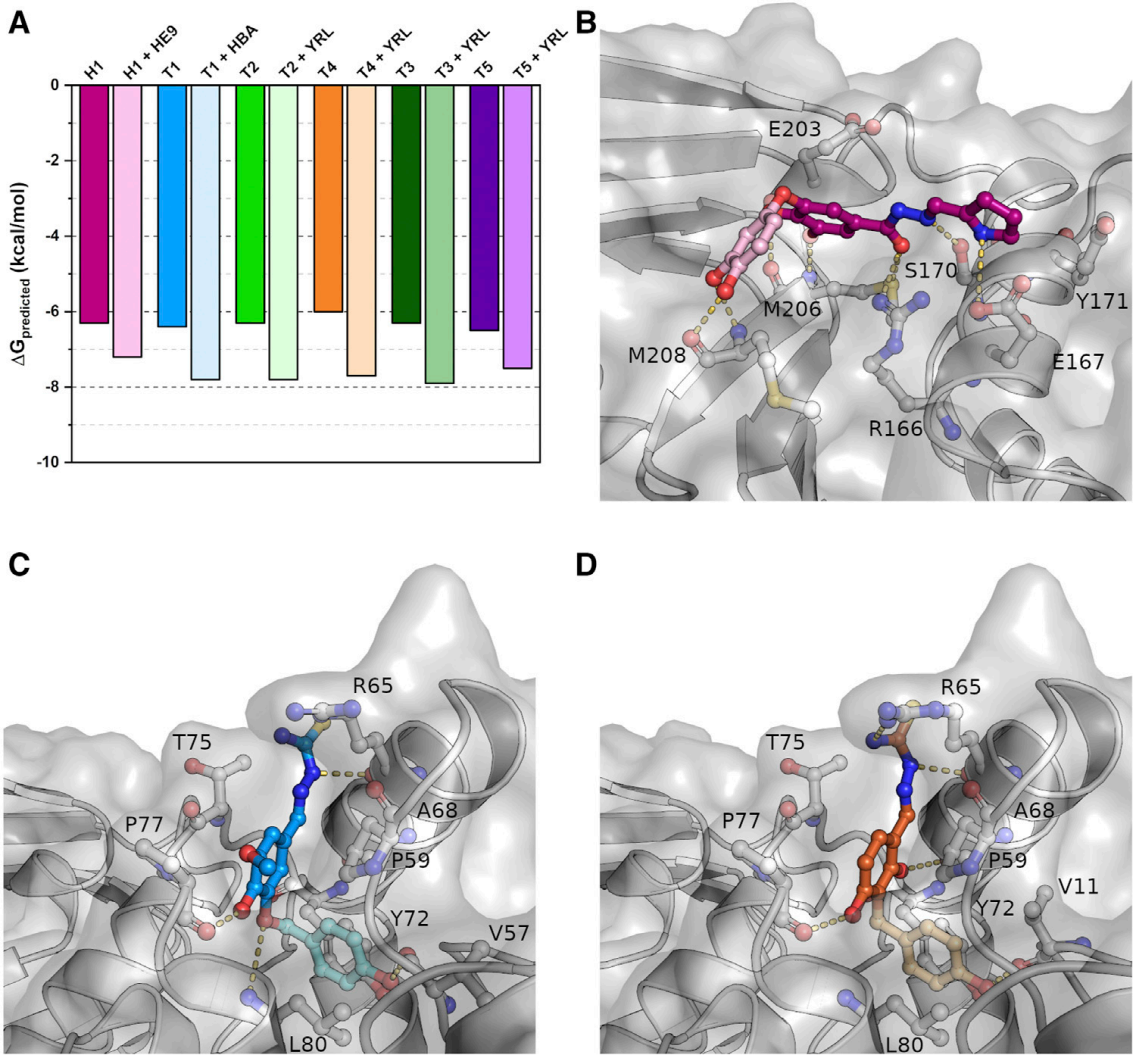
Docking studies of extended compounds highlight potential of binders as lead compounds. **(A)** Results of the docking studies of crystallized and elongated binders. **(B)** The docked compound combining H1 (magenta) with HE9 (PDB: 7ofu, light pink) binding to the S1 site. **(C)** T1 (blue) combined with HBA (PDB: 7oft, light blue) binding to the S2 site. **(D)** T4 (orange) combined with YRL (PDB: 7ofs, light orange) binding to the S2 site. Shown are representative structures of the maximum cluster of the docked conformations.

While most of the compounds do not show a clear inhibitory effect in our assays, their binding positions make them highly valuable candidates in the development of new lead compounds targeting PLpro. To explore the possibilities of compound extension, *in silico* experiments were performed. Here we considered additional PLpro binders from the protein data bank. Among these, three recently described phenolic fragments were further analyzed and included in the compound extensions (Srinivasan et al., 2021), as they were found in adjacent binding positions of the S2 site of SARS-CoV-2 PLpro and show partial overlap with our ligands (Supplementary Figure S5). 4-(2-hydroxyethyl)-phenol (YRL; PDB: 7ofs) and 4-hydroxybenzaldehyde (HBA; PDB: 7oft) bind in a pocket next to the phenol moiety of the thiosemicarbazones T1-T5 (Supplementary Figures S5B, S5C). The *para*-substituent of both phenol derivatives is observed in a position, which is almost identical to a *meta*-methoxy substituent of T1. A similar situation is observed for a *meta*-hydroxyl of H1 related to a symmetry mate of methyl 3,4-dihydroxybenzoate (HE9; PDB: 7ofu) in close proximity to H1 (Supplementary Figure S5A). Molecular docking with either YRL or HBA covalently linked to the specific thiosemicarbazone structures was performed based on the best overlap of these structures, while H1 was elongated with HE9. The resulting docked compounds thereby largely resemble the two experimentally determined binding positions, highlighting the specific interactions of these compounds within their binding pockets, and show an increase of predicted binding energies of 0.8–1.8 kcal/mol relative to the also docked non-extended initial binders (Figure 6A).

For H1 the docking visualizes the high number of possible polar interaction partners within binding distance to the original crystallographic compound position (Figure 6B). In addition to the previously described interactions, the extended compound can further form two hydrogen bonds to the side chain of E167 and main chain carbonyl of E203 due to a 40° rotation around the central carbonyl oxygen relative to the parental compound. In this orientation the newly added phenolic fragment is bound tightly to the protein via two hydrogen bonds between its *meta*- and *para*-hydroxyl group and the main chain carbonyl and amide nitrogen of M208. This suggests that even shallow binding grooves can be useful targets for drug development and opens up possibilities for a variety of polar fragments to be added to the phenol and pyrrole moieties of H1.

Similar to H1, also the thiosemicarbazones in conjunction with the phenolic fragments are predicted to bind tighter inside their binding pocket indicated by lower predicted binding energies. In contrast to hydrazone H1, the compounds are not stabilized by additional hydrogen bonds but mainly by hydrophobic interactions within the binding pocket that is enlarged due to the rotation of side chain L80 (Figures 6C,D). As a result of this movement the extended compounds form new *π*-stacking and *π*-alkyl interactions with the side chains of P59, Y72 and L80. The *para*-hydroxyl group of the added phenolic fragments is further stabilized by a hydrogen bond to the main chain carbonyl of V11 or V57. Differences in binding energies between the thiosemicarbazones, caused by the individual phenolic substitution patterns, were reduced for the compound extension by the addition of the second phenol ring. As a result, the extended compounds will most likely not only possess an increased binding affinity but also an increased inhibitory potential as the separate phenolic fragments alone were already shown to inhibit deubiquitination by PLpro (Srinivasan et al., 2021).

## Discussion

In the search for inhibitors of SARS-CoV-2 PLpro, we performed an X-ray crystallography-based screening. Surprisingly, none of the compounds of our small library of 40 putative zinc coordinating ligands were found to bind at the zinc binding site. Instead we identified six compounds binding to the S1 site (hydrazone H1) or S2 site (thiosemicarbazones T1-T5) of PLpro. These sites function as binding sites for ubiquitin and ISG15 as substrates.

The hydrazone H1 is binding directly at the center of the S1 substrate binding interface, interfering with residues R166/E167 which are highly important for substrate recognition. Mutations at E167, which forms mandatory interactions with both substrates, strongly reduce PLpro activity (Fu et al., 2021; Osipiuk et al., 2021). Binding of H1 has likely a similar effect on E167 as these mutations. Indeed, our biochemical characterization confirms an inhibitory effect of H1 on ubiquitin cleavage by PLpro. At the same time H1 binding to the S1 site does not reduce ISG15 cleavage by PLpro, which might be explained by the reported important interaction of ISG15 with the S2 site of PLpro (Klemm et al., 2020; Osipiuk et al., 2021). As the central role of E167 is not only reported for SARS-CoV-2 but also for SARS-CoV-1 at this site (Békés et al., 2016), the structural features of H1 have the potential to inhibit the deubiquitinase activity of different betacoronaviruses. With differing substrate preferences between the different PLpros (Freitas et al., 2020) it remains to be investigated if H1 can interfere with their specific activity.

The thiosemicarbazones T1-T5 target the S2 binding site. In the binding pocket, the substituents at the phenolic ring of the compounds form a distinct hydrogen bonding pattern exclusively with main chain atoms of the N-terminal helical turn of α3. In contrast to H1 binding at the S1 site, compound binding of T1–T5 to the S2 site of PLpro shows no inhibitory effect. Based on the structural data this might be explained by the smaller overlap of our compounds with natural substrates binding to the S2 site. In addition, with binding affinities of the compounds in the high micromolar range, competitive inhibition will be difficult to detect in our experiments, as the affinity for ISG15 is reported in the lower micromolar range and ubiquitin affinity is approximately 120 µM (Fu et al., 2021; Osipiuk et al., 2021).

Nevertheless the S2 site is highly important for substrate recognition and PLpro activity in general. As T1–T5 are binding to the S2 site at the interface of the Ubl and thumb domain, their thiosemicarbazide moieties interact with residues P77 and T75, which together with V66 are critical for the substrate preferences of SARS-CoV-2 (Shin et al., 2020; Osipiuk et al., 2021). Any mutations of these residues vary the surface properties. In particular size and hydrophobicity of residue 75 alters the second ubiquitin binding site and influences the binding affinity for ISG15 and K48-Ub_2_ (Shin et al., 2020). While SARS-CoV-2 PLpro normally shows a higher efficacy for ISG15 cleavage (Freitas et al., 2020; Klemm et al., 2020; Rut et al., 2020), SARS-CoV-1 with a leucine at this position prefers K48-Ub_2_. Even though showing no inhibition in the present form, binding positions and extensive interactions of the tested compounds represent valuable lead structures for the development of effective inhibitors of PLpro with higher affinity and specificity. Altering the S2 site properties with ligands based on our compounds offers the potential to efficiently slow down the main deubiquitinase activity not only for SARS-CoV-2 but also for other PLpros, as the compounds bind mainly sequence independently to the protein back bone. Therefore, it is reasonable to assume that this type of compound potentially tolerates mutations of the protein, which for example already occurred in the SARS-CoV-2 delta variant (P77L) (Patchett et al., 2021).

**FIGURE 7:**
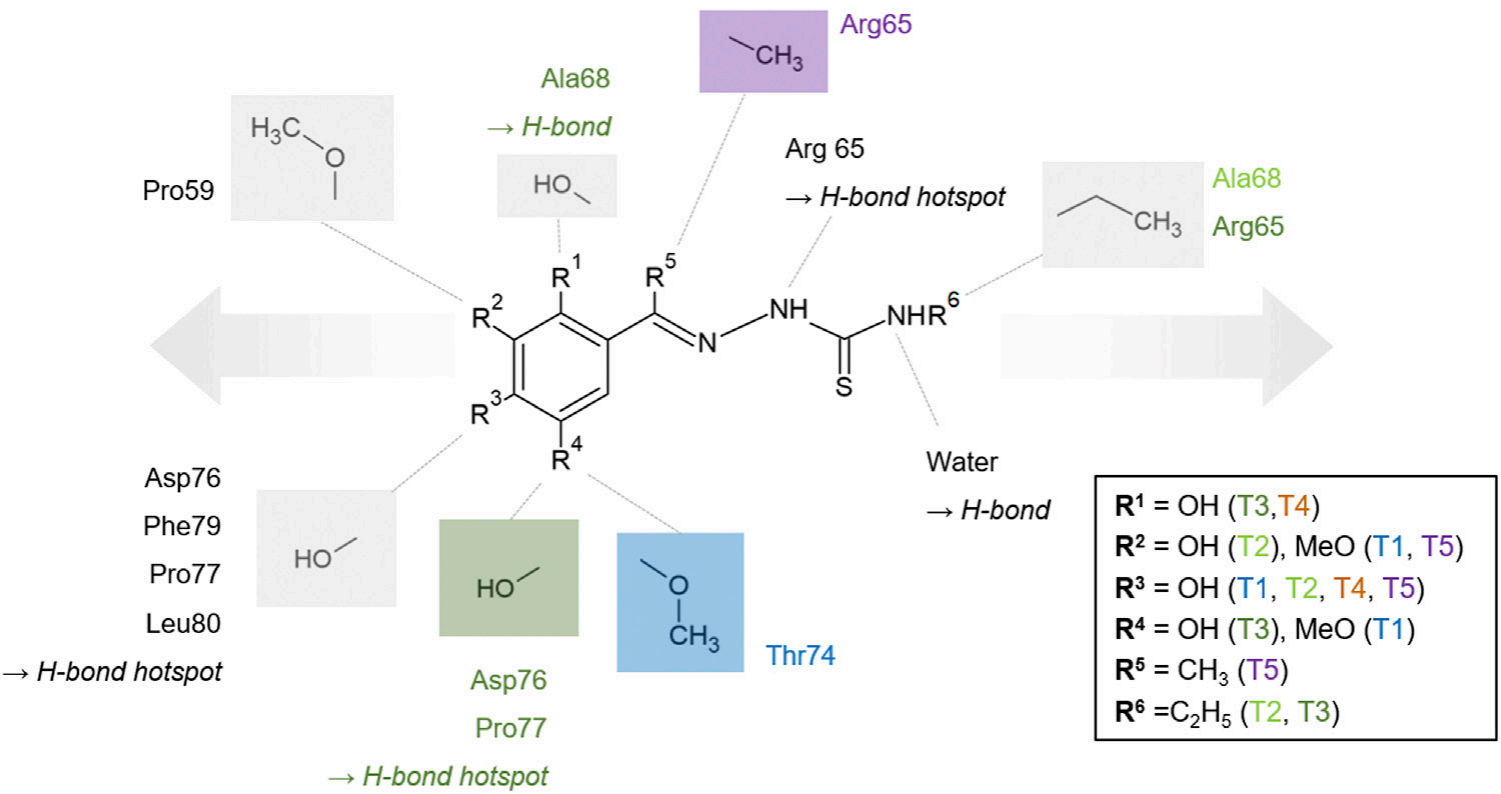
Generic and schematic binding model of the thiosemicarbazones core structure based on the investigated derivatives. Selected PLpro amino acids participating in hydrophobic interaction and hydrogen bonds are displayed and additionally color-coded if they are only relevant for one of the compounds. Conserved and individual hydrogen bond positions are labeled. Moieties, which are exclusively present in one compound, are color-coded, e.g., the methoxylation in the position of R^4^ is exclusive for T1 and colored blue accordingly. The proposed options to extend the thiosemicarbazone compounds, either at the phenyl ring or at the terminal nitrogen of the thiourea moiety, are indicated schematically by arrows.

Our *in silico* approach of extending the initial binders is a first step in structure-based development of novel inhibitors. Combination of such a fragment extension with the multiple options for substitutions at the phenolic rings highlights the potential of developing hydrazones and thiosemicarbazones into potent PLpro inhibitors (Figure 7 and Supplementary Figure S6). The compounds T1-T5 already underline the significance of hydroxylation in *para* position combined with hydroxylation or methoxylation in *meta* position to form multiple hydrogen bonds especially with residues P77 and L80. In addition to the thiosemicarbazide core moiety, which is already involved in hydrogen bonds with R65, further compound optimization can potentially enhance the sterical interference with ISG15. T2 and T3, which are ethyl substituted at this position, represent the first example for extensions at this site and already demonstrated that such a modification does not decrease the binding ability. This position could be further explored by a bulkier substitution to maximize the sterical hindrance of ISG15 binding, which should consistently abolish protein-protein interactions and reduce the deubiquitinase activity of PLpro due to a highly altered binding surface at the S2 site.

The compound interaction hotspots, including the interactions of the polar thiocarbonyl moiety, resemble the modular composition of other previously described thiocarbazone lead compounds (Osmaniye et al., 2021). While thiosemicarbazones currently attract significant interest as anticancer agents (Baruffini et al., 2020), they also show antiviral activity against smallpox and other viruses (Kune, 1964; Rogolino et al., 2015). Hydrazones have shown biological activity for treatment of Alzheimer’s disease, cancer and inflammation with properties rendering them beneficial for medicinal applications (Wahbeh and Milkowski, 2019; de Oliveira Carneiro Brum et al., 2020). These reports can help to increase the pharmacokinetic properties of new designed derivatives based on our lead structures.

Although multiple inhibitors have already been reported for PLpro in different *in vitro* and *in silico* studies, the importance of searching for new inhibitors remains high. Targeting the coronaviral proteases essentially involved in processing the building blocks of the viral transcriptase/replicase complex, continues to be highly attractive (Hilgenfeld, 2014; Dai et al., 2020). Nonetheless, recently published results indicate that some of the previously suggested PLpro inhibitors may lack specificity or optimal pharmaceutical properties (Ma and Wang, 2022). Furthermore, the active site of PLpro does not provide a variety of individual structural features or scaffolds that are in favor for active site drug development. Thus, the identified lead compounds at two different binding sites along with a defined modification strategy are a good starting point to specifically target PLpro deubiquitinase activity and thereby viral replication.

Overall, on the basis of our structural studies, *in vitro* evaluation and *in silico* analysis the described hydrazone and thiosemicarbazone derivatives represent valuable lead compounds targeting the protein-protein interaction of SARS-CoV-2 PLpro. Further investigation of the molecular mechanisms and antiviral properties of improved compounds based on these leads are in progress as the urgent demand for antiviral drugs in the current COVID-19 pandemic remains.

## Data Availability

The crystal structures presented in this study can be found in online repositories. The names of the repository/repositories and accession number(s) can be found below: https://www.rcsb.org/, 7qcg, 7qch, 7qci, 7qcj, 7qck, and 7qcm. Further original contributions presented in the study are included in the article/Supplementary Material, further inquiries can be directed to the corresponding authors.

## References

[B1] Afonine P. V. Poon B. K. Read R. J. Sobolev O. V. Terwilliger T. C. Urzhumtsev A. (2018). Real-space Refinement in PHENIX for Cryo-EM and Crystallography. Acta Cryst. Sect D Struct. Biol. 74, 531–544. 10.1107/S2059798318006551 29872004 PMC6096492

[B2] Báez-Santos Y. M. St. John S. E. Mesecar A. D. (2015). The SARS-Coronavirus Papain-like Protease: Structure, Function and Inhibition by Designed Antiviral Compounds. Antiviral Res. 115, 21–38. 10.1016/j.antiviral.2014.12.015 25554382 PMC5896749

[B3] Bai N. Roder H. Dickson A. Karanicolas J. (2019). Isothermal Analysis of ThermoFluor Data Can Readily Provide Quantitative Binding Affinities. Sci. Rep. 9, 2650. 10.1038/s41598-018-37072-x 30804351 PMC6389909

[B4] Barretto N. Jukneliene D. Ratia K. Chen Z. Mesecar A. D. Baker S. C. (2005). The Papain-like Protease of Severe Acute Respiratory Syndrome Coronavirus Has Deubiquitinating Activity. J. Virol. 79, 15189–15198. 10.1128/JVI.79.24.15189-15198.2005 16306590 PMC1316023

[B5] Barretto N. Jukneliene D. Ratia K. Chen Z. Mesecar A. D. Baker S. C. (2006). Deubiquitinating Activity of the SARS-CoV Papain-like Protease. Adv. Exp. Med. Biol. 581, 37–41. 10.1007/978-0-387-33012-9_5 17037501 PMC7124015

[B6] Baruffini E. Ruotolo R. Bisceglie F. Montalbano S. Ottonello S. Pelosi G. (2020). Mechanistic Insights on the Mode of Action of an Antiproliferative Thiosemicarbazone-Nickel Complex Revealed by an Integrated Chemogenomic Profiling Study. Sci. Rep. 10, 10524. 10.1038/s41598-020-67439-y 32601343 PMC7324377

[B7] Békés M. van der Heden van Noort G. J. Ekkebus R. Ovaa H. Huang T. T. Lima C. D. (2016). Recognition of Lys48-Linked Di-ubiquitin and Deubiquitinating Activities of the SARS Coronavirus Papain-like Protease. Mol. Cel 62, 572–585. 10.1016/j.molcel.2016.04.016 PMC4875570 27203180

[B8] Bosken Y. K. Cholko T. Lou Y.-C. Wu K.-P. Chang C.-e. A. (2020). Insights into Dynamics of Inhibitor and Ubiquitin-like Protein Binding in SARS-CoV-2 Papain-like Protease. Front. Mol. Biosci. 7, 174. 10.3389/fmolb.2020.00174 32850963 PMC7417481

[B9] Burastero O. Niebling S. Defelipe L. A. Günther C. Struve A. Garcia Alai M. M. (2021). eSPC: an Online Data-Analysis Platform for Molecular Biophysics. Acta Cryst. Sect D Struct. Biol. 77, 1241–1250. 10.1107/S2059798321008998 34605428 PMC8489228

[B10] Carcelli M. Rogolino D. Gatti A. De Luca L. Sechi M. Kumar G. (2016). N-acylhydrazone Inhibitors of Influenza Virus PA Endonuclease with Versatile Metal Binding Modes. Sci. Rep. 6, 31500. 10.1038/srep31500 27510745 PMC4980666

[B11] Carcelli M. Rogolino D. Gatti A. Pala N. Corona A. Caredda A. (2017). Chelation Motifs Affecting Metal-dependent Viral Enzymes: N′-acylhydrazone Ligands as Dual Target Inhibitors of HIV-1 Integrase and Reverse Transcriptase Ribonuclease H Domain. Front. Microbiol. 8, 440. 10.3389/fmicb.2017.00440 28373864 PMC5357622

[B12] Chan J. F.-W. Kok K.-H. Zhu Z. Chu H. To K. K.-W. Yuan S. (2020). Genomic Characterization of the 2019 Novel Human-Pathogenic Coronavirus Isolated from a Patient with Atypical Pneumonia after Visiting Wuhan. Emerging Microbes Infections 9, 221–236. 10.1080/22221751.2020.1719902 31987001 PMC7067204

[B13] Chen A. Y. Adamek R. N. Dick B. L. Credille C. V. Morrison C. N. Cohen S. M. (2019). Targeting Metalloenzymes for Therapeutic Intervention. Chem. Rev. 119, 1323–1455. 10.1021/acs.chemrev.8b00201 30192523 PMC6405328

[B14] Citarella A. Scala A. Piperno A. Micale N. (2021). SARS-CoV-2 Mpro: A Potential Target for Peptidomimetics and Small-Molecule Inhibitors. Biomolecules 11, 607. 10.3390/biom11040607 33921886 PMC8073203

[B15] Dai W. Zhang B. Jiang X.-M. Su H. Li J. Zhao Y. (2020). Structure-based Design of Antiviral Drug Candidates Targeting the SARS-CoV-2 Main Protease. Science 368, 1331–1335. 10.1126/science.abb4489 32321856 PMC7179937

[B16] de Oliveira Carneiro Brum J. França T. C. C. LaPlante S. R. Villar J. D. F. (2020). Synthesis and Biological Activity of Hydrazones and Derivatives: A Review. Mini. Rev. Med. Chem. 20, 342–368. 10.2174/1389557519666191014142448 31612828

[B17] de Wit E. van Doremalen N. Falzarano D. Munster V. J. (2016). SARS and MERS: Recent Insights into Emerging Coronaviruses. Nat. Rev. Microbiol. 14, 523–534. 10.1038/nrmicro.2016.81 27344959 PMC7097822

[B18] Emsley P. Cowtan K. (2004). Coot: Model-Building Tools for Molecular Graphics. Acta Crystallogr. D Biol. Cryst. 60, 2126–2132. 10.1107/S0907444904019158 15572765

[B19] Freitas B. T. Durie I. A. Murray J. Longo J. E. Miller H. C. Crich D. (2020). Characterization and Noncovalent Inhibition of the Deubiquitinase and deISGylase Activity of SARS-CoV-2 Papain-like Protease. ACS Infect. Dis. 6, 2099–2109. 10.1021/acsinfecdis.0c00168 32428392

[B20] Fu Z. Huang B. Tang J. Liu S. Liu M. Ye Y. (2021). The Complex Structure of GRL0617 and SARS-CoV-2 PLpro Reveals a Hot Spot for Antiviral Drug Discovery. Nat. Commun. 12, 488. 10.1038/s41467-020-20718-8 33473130 PMC7817691

[B21] Günther S. Reinke P. Y. A. Fernández-García Y. Lieske J. Lane T. J. Ginn H. M. (2021). X-ray Screening Identifies Active Site and Allosteric Inhibitors of SARS-CoV-2 Main Protease. Science 372, 642–646. 10.1126/science.abf7945 33811162 PMC8224385

[B22] Hilgenfeld R. (2014). From SARS to MERS: Crystallographic Studies on Coronaviral Proteases Enable Antiviral Drug Design. FEBS J. 281, 4085–4096. 10.1111/febs.12936 25039866 PMC7163996

[B23] Honorato R. V. Koukos P. I. Jiménez-García B. Tsaregorodtsev A. Verlato M. Giachetti A. (2021). Structural Biology in the Clouds: The WeNMR-EOSC Ecosystem. Front. Mol. Biosci. 8, 708. 10.3389/fmolb.2021.729513 PMC8356364 34395534

[B24] Hsu D. C. Roth H. S. West D. C. Botham R. C. Novotny C. J. Schmid S. C. (2012). Parallel Synthesis and Biological Evaluation of 837 Analogues of Procaspase-Activating Compound 1 (PAC-1). ACS Comb. Sci. 14, 44–50. 10.1021/co2001372 22007686 PMC3253983

[B25] Huan L. C. Anh D. T. Hai P.-T. Anh L. D. Park E. J. Ji A. Y. (2020). Design, Synthesis, and Evaluation of Novel N'-substituted-1-(4-chlorobenzyl)-1H-indol-3-carbohydrazides as Antitumor Agents. J. Enzyme Inhib. Med. Chem. 35, 1854–1865. 10.1080/14756366.2020.1816997 32981382 PMC7534272

[B26] Hunter J. D. (2007). Matplotlib: A 2D Graphics Environment. Comput. Sci. Eng. 9, 90–95. 10.1109/MCSE.2007.55

[B27] Klemm T. Ebert G. Calleja D. J. Allison C. C. Richardson L. W. Bernardini J. P. (2020). Mechanism and Inhibition of the Papain‐like Protease, PLpro, of SARS‐CoV‐2. EMBO J. 39, e106275. 10.15252/embj.2020106275 32845033 PMC7461020

[B28] Kune G. A. (1964). To-Day’s Drugs: Methisazone. Br. Med. J. 2, 621. 14171075 PMC1816639

[B29] Kurkcuoglu Z. Koukos P. I. Citro N. Trellet M. E. Rodrigues J. P. G. L. M. Moreira I. S. (2018). Performance of HADDOCK and a Simple Contact-Based Protein-Ligand Binding Affinity Predictor in the D3R Grand Challenge 2. J. Comput. Aided Mol. Des. 32, 175–185. 10.1007/s10822-017-0049-y 28831657 PMC5767195

[B30] Liebschner D. Afonine P. V. Baker M. L. Bunkóczi G. Chen V. B. Croll T. I. (2019). Macromolecular Structure Determination Using X-Rays, Neutrons and Electrons: Recent Developments in Phenix. Acta Cryst. Sect D Struct. Biol. 75, 861–877. 10.1107/S2059798319011471 31588918 PMC6778852

[B31] Liu G. Lee J.-H. Parker Z. M. Acharya D. Chiang J. J. van Gent M. (2021). ISG15-dependent Activation of the Sensor MDA5 Is Antagonized by the SARS-CoV-2 Papain-like Protease to Evade Host Innate Immunity. Nat. Microbiol. 6, 467–478. 10.1038/s41564-021-00884-1 33727702 PMC8103894

[B32] Lu R. Zhao X. Li J. Niu P. Yang B. Wu H. (2020). Genomic Characterisation and Epidemiology of 2019 Novel Coronavirus: Implications for Virus Origins and Receptor Binding. Lancet 395, 565–574. 10.1016/S0140-6736(20)30251-8 32007145 PMC7159086

[B33] Ma C. Wang J. (2022). Validation and Invalidation of SARS-CoV-2 Papain-like Protease Inhibitors. ACS Pharmacol. Transl. Sci. 5, 102–109. 10.1021/acsptsci.1c00240 35178512 PMC8806001

[B34] McKinney W. (2010). “Data Structures for Statistical Computing in Python,” in Proceedings of the 9th Python in Science Conference, Austin, Texas, 28.06-03.07, 56–61. 10.25080/Majora-92bf1922-00a

[B35] Mevissen T. E. T. Komander D. (2017). Mechanisms of Deubiquitinase Specificity and Regulation. Annu. Rev. Biochem. 86, 159–192. 10.1146/annurev-biochem-061516-044916 28498721

[B36] Moriarty N. W. Grosse-Kunstleve R. W. Adams P. D. (2009). Electronic Ligand Builder and Optimization Workbench(eLBOW): a Tool for Ligand Coordinate and Restraint Generation. Acta Crystallogr. D Biol. Cryst. 65, 1074–1080. 10.1107/S0907444909029436 19770504 PMC2748967

[B37] Morris G. M. Goodsell D. S. Halliday R. S. Huey R. Hart W. E. Belew R. K. (1998). Automated Docking Using a Lamarckian Genetic Algorithm and an Empirical Binding Free Energy Function. J. Comput. Chem. 19, 1639–1662. 10.1002/(SICI)1096-987X(19981115)19:14<1639::AID-JCC10>3.0.CO;2-B

[B38] Morris G. M. Huey R. Lindstrom W. Sanner M. F. Belew R. K. Goodsell D. S. (2009). AutoDock4 and AutoDockTools4: Automated Docking with Selective Receptor Flexibility. J. Comput. Chem. 30, 2785–2791. 10.1002/jcc.21256 19399780 PMC2760638

[B39] Munnur D. Teo Q. Eggermont D. Lee H. H. Y. Thery F. Ho J. (2021). Altered ISGylation Drives Aberrant Macrophage-dependent Immune Responses during SARS-CoV-2 Infection. Nat. Immunol. 22, 1416–1427. 10.1038/s41590-021-01035-8 34663977

[B40] Niebling S. Burastero O. Bürgi J. Günther C. Defelipe L. A. Sander S. (2021). FoldAffinity: Binding Affinities from nDSF Experiments. Sci. Rep. 11, 9572. 10.1038/s41598-021-88985-z 33953265 PMC8099913

[B41] Oliphant T. (2006). Guide to NumPy. USA: Tregol Publishing.

[B42] Osipiuk J. Wydorski P. M. Lanham B. T. Tesar C. Endres M. Engle E. (2021). Dual Domain Recognition Determines SARS-CoV-2 PLpro Selectivity for Human ISG15 and K48-Linked Di-ubiquitin. bioRxiv. [Preprint]. 10.1101/2021.09.15.460543 PMC10126577 37185902

[B43] Osmaniye D. Kurban B. Sağlık B. N. Levent S. Özkay Y. Kaplancıklı Z. A. (2021). Novel Thiosemicarbazone Derivatives: *In Vitro* and *In Silico* Evaluation as Potential MAO-B Inhibitors. Molecules 26, 6640. 10.3390/molecules26216640 34771054 PMC8587871

[B44] Panchariya L. Khan W. A. Kuila S. Sonkar K. Sahoo S. Ghoshal A. (2021). Zinc2+ Ion Inhibits SARS-CoV-2 Main Protease and Viral Replication *In Vitro* . Chem. Commun. 57, 10083–10086. 10.1039/D1CC03563K 34514483

[B45] Patchett S. Lv Z. Rut W. Békés M. Drag M. Olsen S. K. (2021). A Molecular Sensor Determines the Ubiquitin Substrate Specificity of SARS-CoV-2 Papain-like Protease. Cel Rep. 36, 109754. 10.1016/j.celrep.2021.109754 PMC8423903 34547223

[B46] Pearce N. M. Krojer T. Bradley A. R. Collins P. Nowak R. P. Talon R. (2017). A Multi-crystal Method for Extracting Obscured Crystallographic States from Conventionally Uninterpretable Electron Density. Nat. Commun. 8, 15123. 10.1038/ncomms15123 28436492 PMC5413968

[B47] Ratia K. Saikatendu K. S. Santarsiero B. D. Barretto N. Baker S. C. Stevens R. C. (2006). Severe Acute Respiratory Syndrome Coronavirus Papain-like Protease: Structure of a Viral Deubiquitinating Enzyme. Proc. Natl. Acad. Sci. 103, 5717–5722. 10.1073/pnas.0510851103 16581910 PMC1458639

[B48] Rogolino D. Bacchi A. De Luca L. Rispoli G. Sechi M. Stevaert A. (2015). Investigation of the Salicylaldehyde Thiosemicarbazone Scaffold for Inhibition of Influenza Virus PA Endonuclease. J. Biol. Inorg. Chem. 20, 1109–1121. 10.1007/s00775-015-1292-0 26323352

[B49] Rut W. Lv Z. Zmudzinski M. Patchett S. Nayak D. Snipas S. J. (2020). Activity Profiling and crystal Structures of Inhibitor-Bound SARS-CoV-2 Papain-like Protease: A Framework for Anti-COVID-19 Drug Design. Sci. Adv. 6, eabd4596. 10.1126/sciadv.abd4596 33067239 PMC7567588

[B50] Sargsyan K. Lin C.-C. Chen T. Grauffel C. Chen Y.-P. Yang W.-Z. (2020). Multi-targeting of Functional Cysteines in Multiple Conserved SARS-CoV-2 Domains by Clinically Safe Zn-Ejectors. Chem. Sci. 11, 9904–9909. 10.1039/D0SC02646H 34094251 PMC8162115

[B51] Schechter I. Berger A. (1967). On the Size of the Active Site in Proteases. I. Papain. Biochem. Biophys. Res. Commun. 27, 157–162. 10.1016/s0006-291x(67)80055-x 6035483

[B52] Shen Z. Ratia K. Cooper L. Kong D. Lee H. Kwon Y. (2021). Design of SARS-CoV-2 PLpro Inhibitors for COVID-19 Antiviral Therapy Leveraging Binding Cooperativity. J. Med. Chem. 65, 2940–2955. 10.1021/acs.jmedchem.1c01307 34665619 PMC8547495

[B53] Shin D. Mukherjee R. Grewe D. Bojkova D. Baek K. Bhattacharya A. (2020). Papain-like Protease Regulates SARS-CoV-2 Viral Spread and Innate Immunity. Nature 587, 657–662. 10.1038/s41586-020-2601-5 32726803 PMC7116779

[B54] Srinivasan V. Brognaro H. Prabhu P. R. Souza E. E. D. Günther S. Reinke P. Y. A. (2021). SARS-CoV-2 Papain-like Protease PLpro in Complex with Natural Compounds Reveal Allosteric Sites for Antiviral Drug Design. bioRxiv. [Preprint]. 10.1101/2021.11.17.468943

[B55] Studier F. W. (2005). Protein Production by Auto-Induction in High-Density Shaking Cultures. Protein Expr. Purif. 41, 207–234. 10.1016/j.pep.2005.01.016 15915565

[B56] te Velthuis A. J. W. van den Worm S. H. E. Sims A. C. Baric R. S. Snijder E. J. van Hemert M. J. (2010). Zn^2+^ Inhibits Coronavirus and Arterivirus RNA Polymerase Activity *In Vitro* and Zinc Ionophores Block the Replication of These Viruses in Cell Culture. Plos Pathog. 6, e1001176. 10.1371/journal.ppat.1001176 21079686 PMC2973827

[B57] Tregoning J. S. Flight K. E. Higham S. L. Wang Z. Pierce B. F. (2021). Progress of the COVID-19 Vaccine Effort: Viruses, Vaccines and Variants versus Efficacy, Effectiveness and Escape. Nat. Rev. Immunol. 21, 626–636. 10.1038/s41577-021-00592-1 34373623 PMC8351583

[B58] V’kovski P. Kratzel A. Steiner S. Stalder H. Thiel V. (2020). Coronavirus Biology and Replication: Implications for SARS-CoV-2. Nat. Rev. Microbiol. 19, 155–170. 10.1038/s41579-020-00468-6 33116300 PMC7592455

[B59] Vabret N. Britton G. J. Gruber C. Hegde S. Kim J. Kuksin M. (2020). Immunology of COVID-19: Current State of the Science. Immunity 52, 910–941. 10.1016/j.immuni.2020.05.002 32505227 PMC7200337

[B60] van der Walt S. Colbert S. C. Varoquaux G. (2011). The NumPy Array: A Structure for Efficient Numerical Computation. Comput. Sci. Eng. 13, 22–30. 10.1109/MCSE.2011.37

[B61] van Zundert G. C. P. Rodrigues J. P. G. L. M. Trellet M. Schmitz C. Kastritis P. L. Karaca E. (2016). The HADDOCK2.2 Web Server: User-Friendly Integrative Modeling of Biomolecular Complexes. J. Mol. Biol. 428, 720–725. 10.1016/j.jmb.2015.09.014 26410586

[B62] Vangone A. Schaarschmidt J. Koukos P. Geng C. Citro N. Trellet M. E. (2019). Large-scale Prediction of Binding Affinity in Protein-Small Ligand Complexes: the PRODIGY-LIG Web Server. Bioinformatics 35, 1585–1587. 10.1093/bioinformatics/bty816 31051038

[B63] Virtanen P. Gommers R. Oliphant T. E. Haberland M. Reddy T. Cournapeau D. (2020). SciPy 1.0: Fundamental Algorithms for Scientific Computing in Python. Nat. Methods 17, 261–272. 10.1038/s41592-019-0686-2 32015543 PMC7056644

[B64] Wahbeh J. Milkowski S. (2019). The Use of Hydrazones for Biomedical Applications. SLAS Techn. 24, 161–168. 10.1177/2472630318822713 30744468

[B65] WHO (2021). WHO Coronavirus (COVID-19) Dashboard. Available at: https://covid19.who.int (Accessed November 27, 2021).

[B66] Winter G. Waterman D. G. Parkhurst J. M. Brewster A. S. Gildea R. J. Gerstel M. (2018). DIALS: Implementation and Evaluation of a New Integration Package. Acta Cryst. Sect D Struct. Biol. 74, 85–97. 10.1107/S2059798317017235 29533234 PMC5947772

[B67] Zhang L. Lin D. Sun X. Curth U. Drosten C. Sauerhering L. (2020). Crystal Structure of SARS-CoV-2 Main Protease Provides a Basis for Design of Improved α-ketoamide Inhibitors. Science 368, 409–412. 10.1126/science.abb3405 32198291 PMC7164518

[B68] Zhou P. Yang X.-L. Wang X.-G. Hu B. Zhang L. Zhang W. (2020). A Pneumonia Outbreak Associated with a New Coronavirus of Probable Bat Origin. Nature 579, 270–273. 10.1038/s41586-020-2012-7 32015507 PMC7095418

[B69] Zmudzinski M. Rut W. Olech K. Granda J. Giurg M. Burda-Grabowska M. (2020). Ebselen Derivatives Are Very Potent Dual Inhibitors of SARS-CoV-2 Proteases - PLpro and Mpro in *In Vitro* Studies. bioRxiv. [Preprint]. 10.1101/2020.08.30.273979

