# Supplementary material for: Hydrazones and Thiosemicarbazones Targeting Protein-Protein-Interactions of SARS-CoV-2 Papain-like Protease
Source: Front Chem. 2022 Apr 11;10:832431. doi: 10.3389/fchem.2022.832431 (PMC9038201; doi:10.3389/fchem.2022.832431)
Supplement: Supplementary file 1 [file DataSheet1.pdf]

Supplementary Material

Hydrazones and Thiosemicarbazones Targeting Protein-Protein-Interactions of SARS-CoV-2 Papain-like Protease

Wiebke Ewert[1,*], Sebastian Günther[1,*], Francesca Miglioli[2], Sven Falke[1], Patrick Y. A. Reinke[1], Stephan Niebling[3], Christian Günther[3], Huijong Han[4], Vasundara Srinivasan[5], Hévila Brognaro[5], Julia Lieske[1], Kristina Lorenzen[4], Maria M. Garcia-Alai[3], Christian Betzel[5], Mauro Carcelli[2], Winfried Hinrichs[6], Dominga Rogolino[2] and Alke Meents[1]

[1] Center for Free-Electron Laser Science CFEL, Deutsches Elektronen-Synchrotron DESY, Hamburg, Germany

[2] Department of Chemistry, Life Sciences and Environmental Sustainability, University of Parma, Parma, Italy

[3] European Molecular Biology Laboratory Hamburg, DESY, Hamburg, Germany

[4] European XFEL GmbH, Schenefeld, Germany

[5] Institute of Biochemistry and Molecular Biology, Laboratory for Structural Biology of Infection and Inflammation, Department of Chemistry, University Hamburg, Hamburg, Germany

[6] Institute of Biochemistry, University Greifswald, Greifswald, Germany

*** Correspondence:**
Corresponding Author
wiebke.ewert@desy.de, sebastian.guenther@desy.de

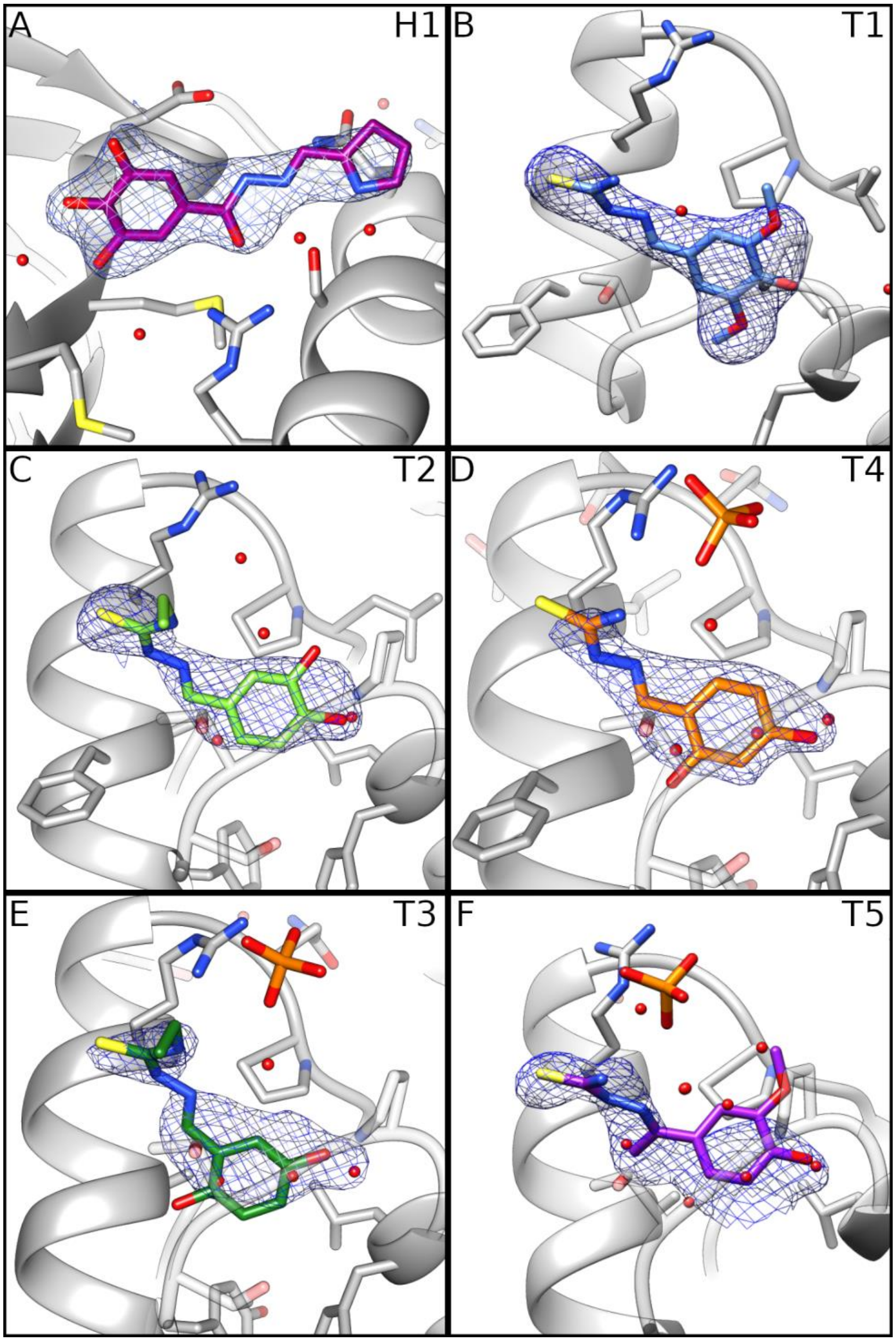

Figure S1: PanDDA maps of the binding hydrazone (H1) and thiosemicarbazone (T1-T5) compounds. Compounds H1 and T1 were refined with an occupancy of 1, while residues T2-T5 were refined with occupancies between 0.42-0.57.

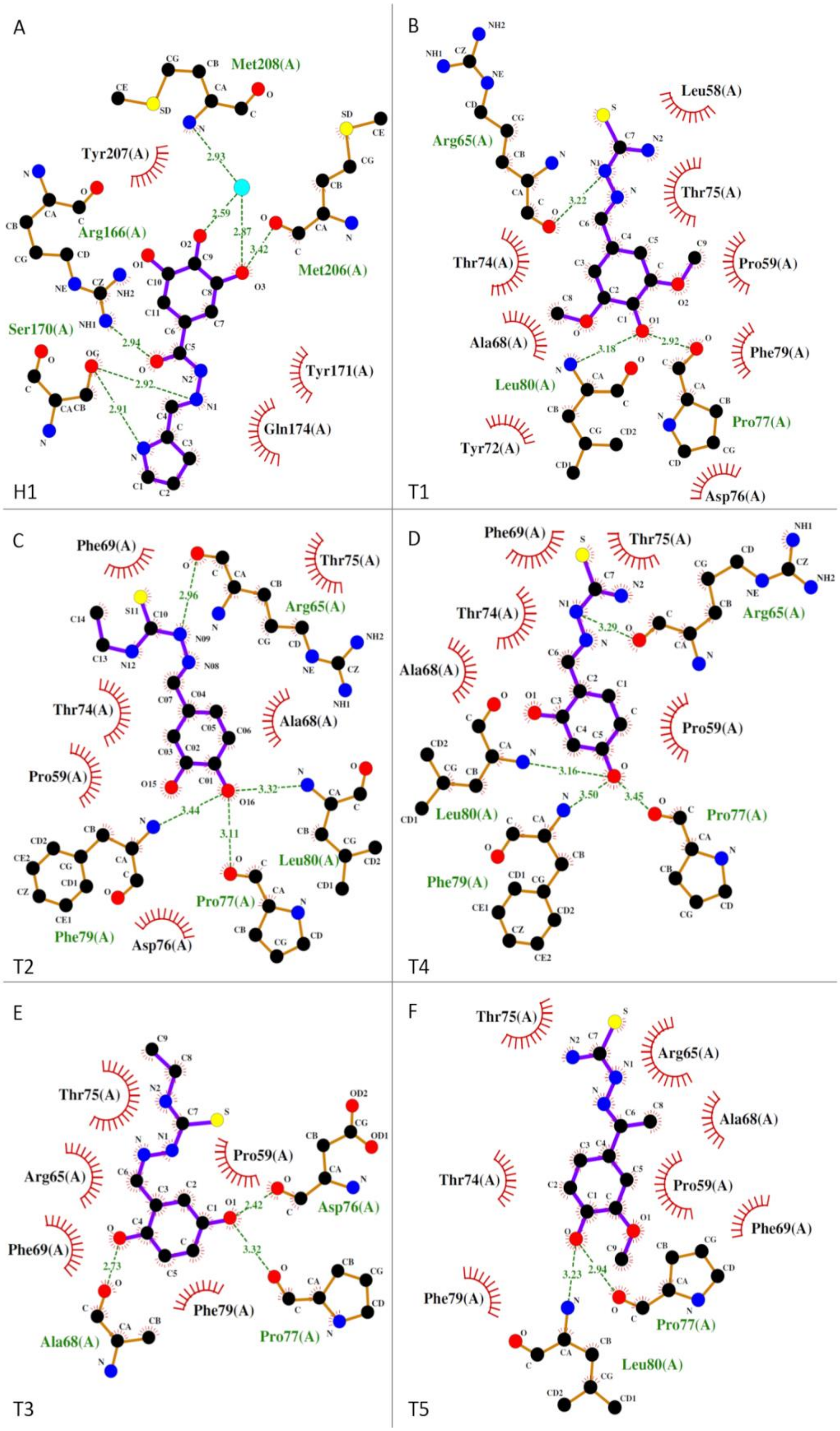

Figure S2: 2D interaction diagrams of the hydrazone and thiosemicarbazones bound to PLpro. Plots were generated using LigPlot+ V2.2 (EMBL-EBI).

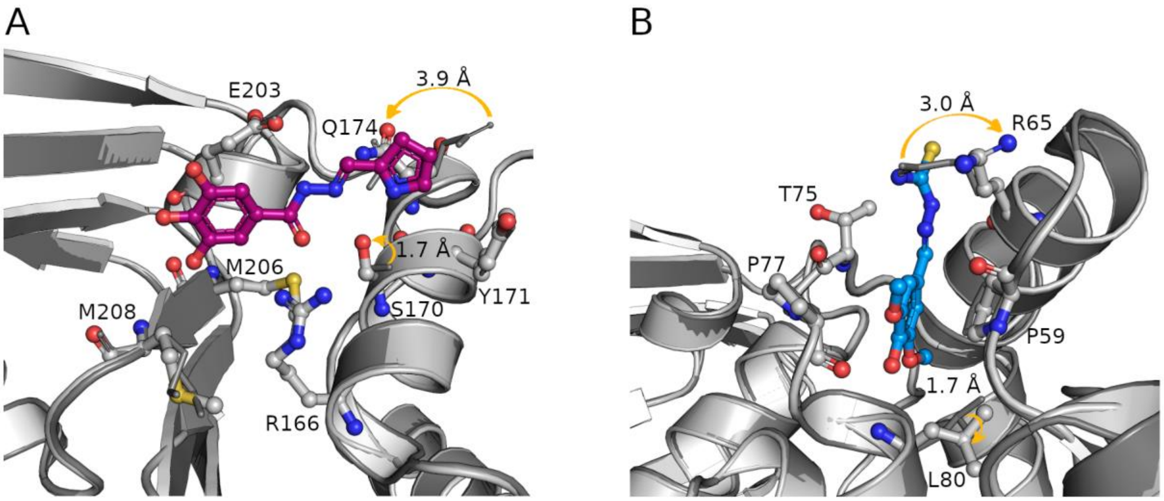

Figure S3: Hydrazone and thiosemicarbazones induce several side chain rearrangements upon binding (indicated by arrow labeled with displacement values). Compound bound PLpro is shown in light grey, while the ligand-free enzyme (PDB: 7nfv) is represented in dark grey with smaller stick side chains. (A) The pyrrole and hydrazone moiety of H1 form hydrogen bonds inducing conformational changes of the side chains of S170 and Q174. (B) The thiosemicarbazone T1 induces conformational changes of the side chains of residues R65 and L80, which results in an extended binding pocket.

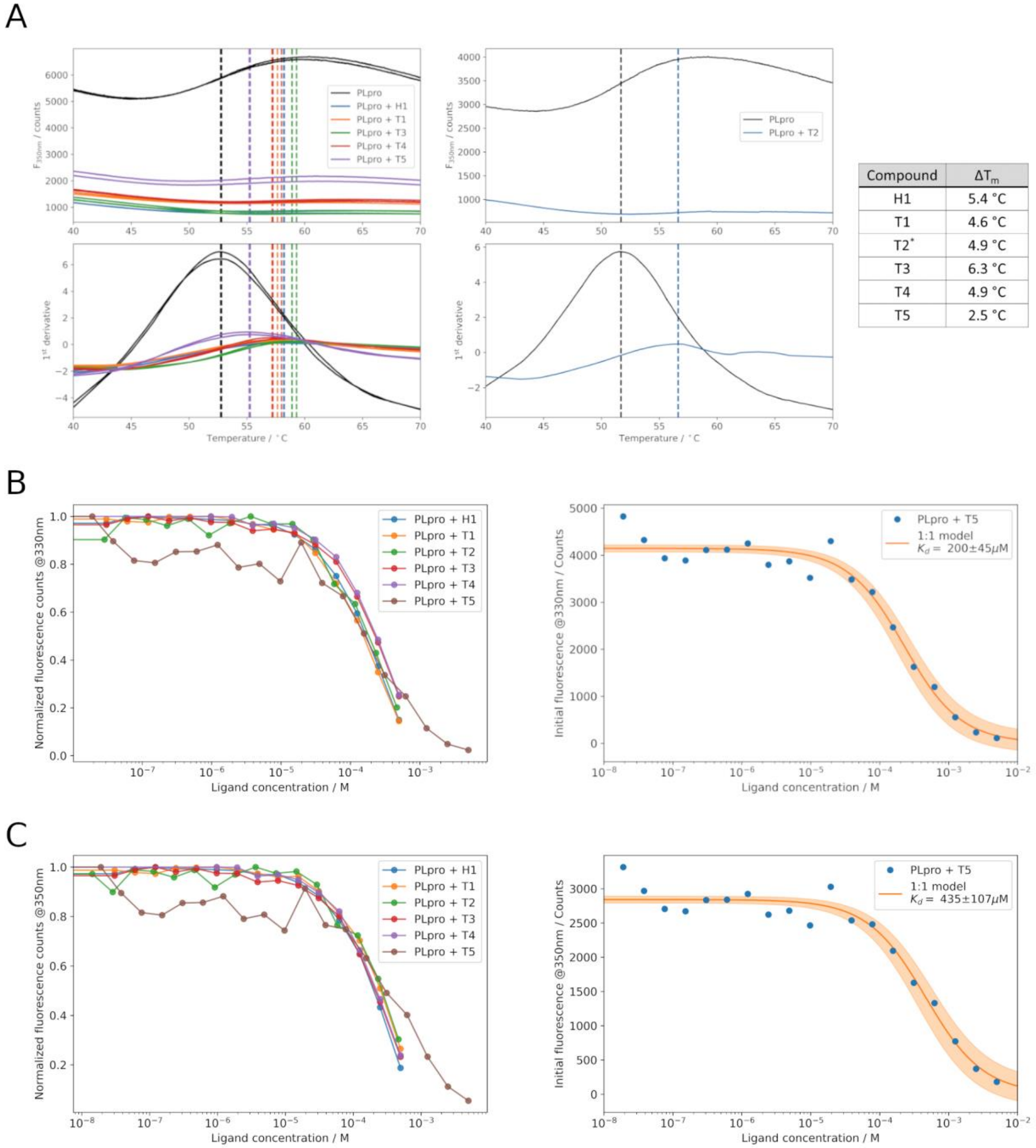

Figure S4: Nano DSF data. (A) Initial binding check by nDSF using 54 µM PLpro with 500 µM of each ligand. PLpro melting temperature was 52.8 °C. For T2 (marked with an asterisk) the test was ran with a different PLpro batch with a melting temperature of 51.7 °C, therefore, it is shown in a separate graph. (B) Fluorescence titration at 330 nm for PLpro and T5 and comparison with other ligands. (C) Fluorescence titration at 350 nm for PLpro and T5 and comparison with other ligands.

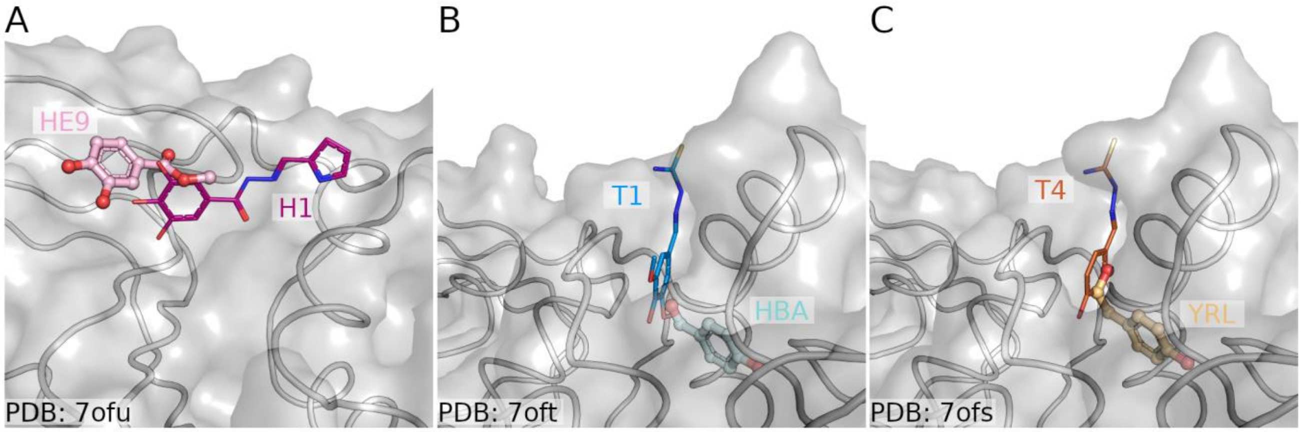

Figure S5: Structural comparison of recently described phenolic fragments and the new found hydrazone and thiosemicarbazones binding in close proximity. (A) Overlap of H1 (magenta) with HE9 (PDB: 7ofu, light pink) binding to the S1 site. The position of HE9 is inferred through crystallographic symmetry from the original structure where it was placed close to the S2 site. (B+C) T1 (blue) and T4 (orange) are examples for the thiosemicarbazones overlapping with HBA (PDB: 7oft, light blue) and YRL (PDB: 7ofs, light orange) binding to the S2 site.

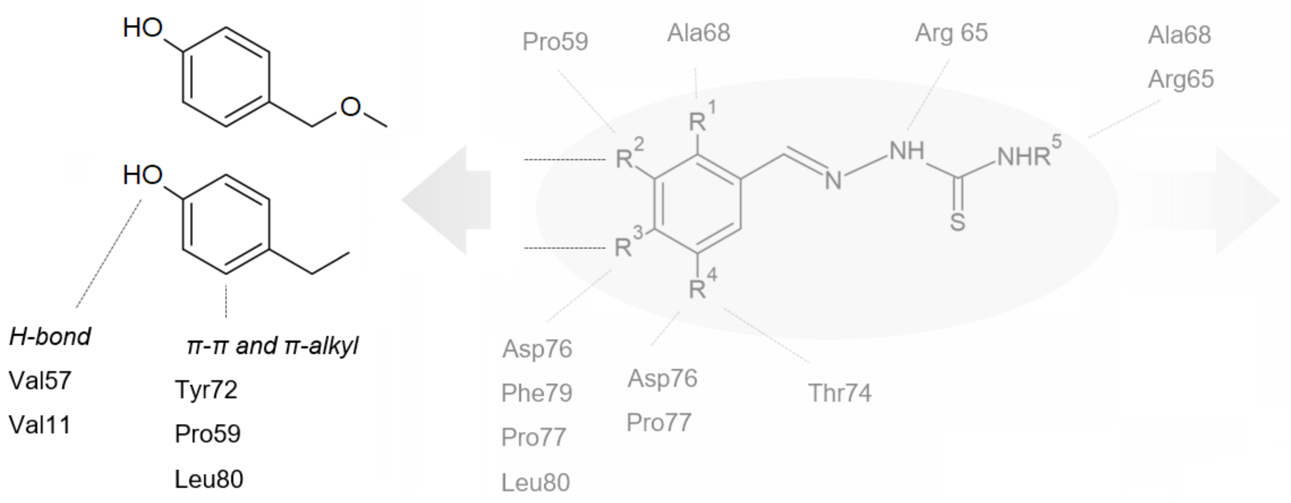

Figure S6: Generic and schematic binding model of the thiosemicarbazone lead structure extension as investigated by *in silico* docking and complementing the information provided by Figure 7. The binding mode of the thiosemicarbazones extended by a phenolic moiety is displayed in more detail in Figure 6C/D for T1 and T4 respectively. The phenol ring, which is linked to the aromatic ring of the core structure at position R^2 or R^3 either with (T1+ HBA) or without (T2-T5 + YRL) an ether function (Table S3), is contributing to π-π and π-alkyl interactions with the protein and the *para*-hydroxyl group allows additional hydrogen bonding as specified.

Table S1: List of the screened compounds.

Quinolone derivatives

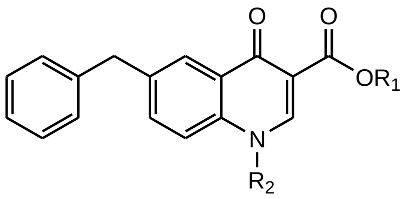

R1 =H	R2 =H	**(1)**
R1 = H	R2 = CH_2CH_2OH	**(2)**
R1 = H	R2 = CH_2Ph	**(3)**
R1 = CH_2CH_3	R2 = CH_2Ph	**(4)**

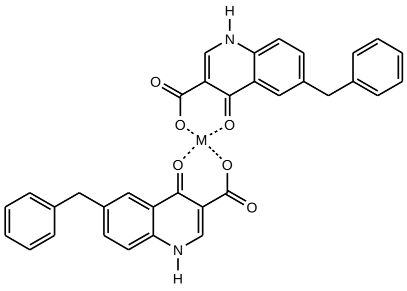

M = Mg^{2+}	**(5)**
M = Mn^{2+}	**(6)**

(Bacchi et al., 2011)

Thiosemicarbazones

Isatin thiosemicarbazones

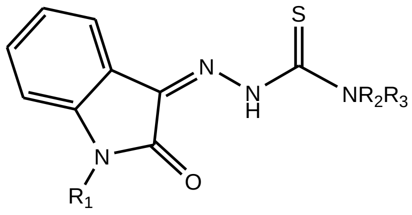

R1 =H	R2 =H	R3 =H	**(7)**
R1 =H	R2 = H	R3 = CH_2CH_3	**(8)**
R1 = H	R2 = CH_3	R3 = CH_3	**(9)**
R1 = CH_3	R2 = H	R3 =H	**(10)**
R1 = CH_3	R2 = H	R3 = CH_2CH_3	**(11)**
R1 = CH_3	R2 = CH_3	R3 = CH_3	**(12)**

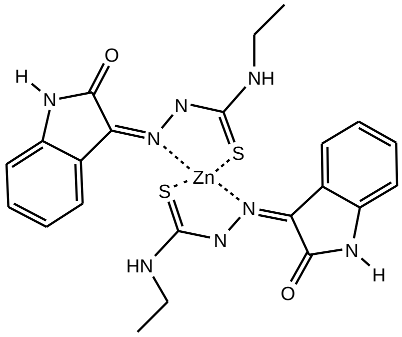

(Chen et al., 2005)

(13), unpublished results

Hydroxylated phenyl thiosemicarbazones

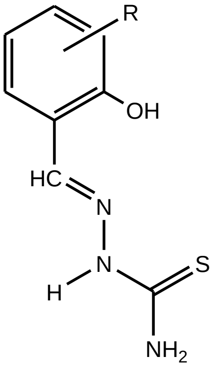

R = 3-methoxy		**(14)**
R = 3-hydroxy		**(15)**
R = 4-hydroxy		**(16, T4)**
R = 5-hydroxy		**(17)**
R = 5-methoxy		**(18)**

(Rogolino et al., 2015)

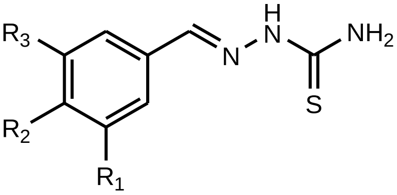

R1 = H	R2 =OH	R3 = OH	**(19)**
R1 = OCH_3	R2 = OH	R3 = OCH_3	**(20, T1)**

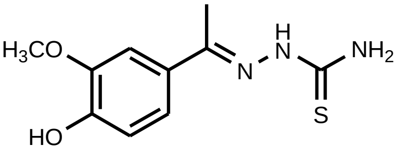

(21, T5)

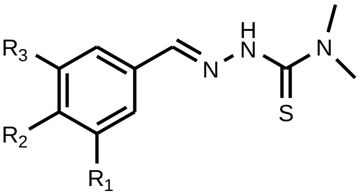

R1 = OH	R2 = OH	R3 = OH	**(22)**

R1 = H	R2 = OCH_3	R3 = OCH_3	**(23)**
R1 = H	R2 = H	R3 = H	**(24)**

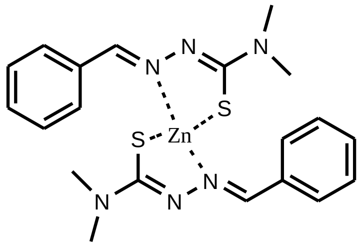

(25), unpublished results

(Rogolino et al., 2017)

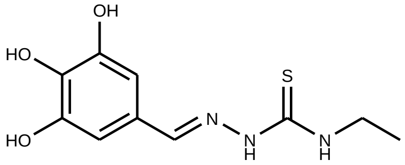

(26)

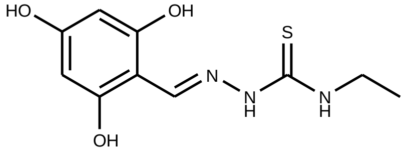

(27)

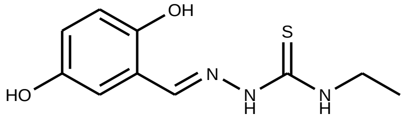

(28, T3)

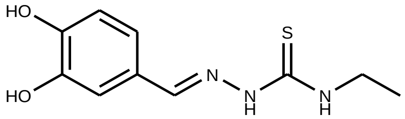

(29, T2)

(Carcelli et al., 2020)

Hydroxyquinoline derivatives

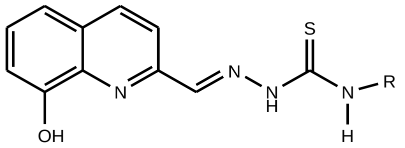

R = H **(30)**

R = CH_2CH_3 **(31)**

(Rogolino et al., 2017)

Hydroxylated hydrazones

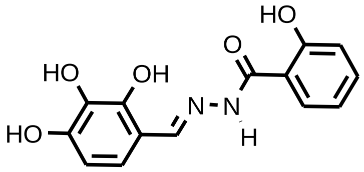

(32)

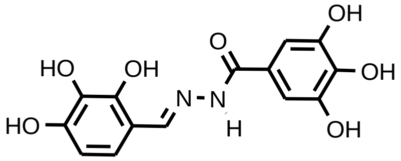

(33)

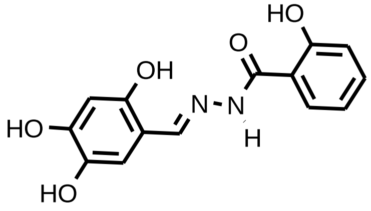

(34)

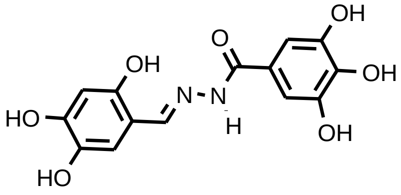

(35)

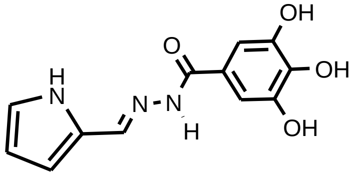

(36, H1)

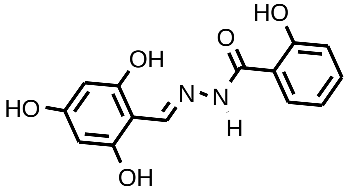

(37)

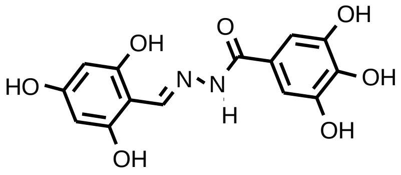

(38)

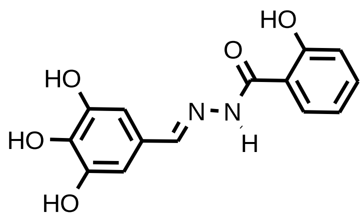

(39)

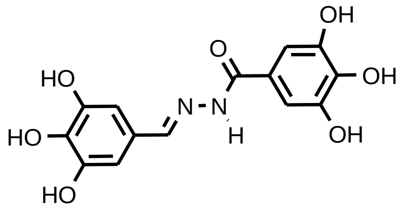

(40)

(Carcelli et al., 2016)

Table S2: Data collection and refinement statistics.

**Compound**	**H1**	**T1**	**T2**	**T3**	**T4**	**T5**
**PDB ID**	7QCG	7QCH	7QCI	7QCK	7QCJ	7QCM
**Data Collection**						
Space group	P 3_2 2 1	P 3_2 2 1	P 3_2 2 1	P 3_2 2 1	P 3_2 2 1	P 3_2 2 1
Cell dimensions						
a, b, c (Å)	83.21 83.21 134.03	83.65 83.65 134.17	83.37 83.37 133.89	83.49 83.49 134.47	83.37 83.37 134.24	83.06 83.06 134.06
α, β, γ (°)	90 90 120	90 90 120	90 90 120	90 90 120	90 90 120	90 90 120
Resolution (Å)	63.47 - 1.75 (1.813 - 1.75)	41.82 - 1.88 (1.947 - 1.88)	39.8 - 1.76 (1.823 - 1.76)	49.24 - 1.84 (1.906 - 1.84)	49.16 - 1.92 (1.989 - 1.92)	44.69 - 1.77 (1.833 - 1.77)
No. reflections	54802 (5451)	44773 (4402)	53973 (5303)	47706 (4711)	41853 (4127)	52772 (5173)
R_{merge}	0.077 (0.872)	0.073 (0.785)	0.077 (0.928)	0.096 (0.878)	0.113 (0.848)	0.083 (0.855)
R_{meas}	0.081 (0.915)	0.076 (0.827)	0.081 (0.975)	0.101 (0.924)	0.118 (0.894)	0.087 (0.897)
R_{pim}	0.025 (0.276)	0.023 (0.261)	0.025 (0.295)	0.031 (0.284)	0.036 (0.281)	0.027 (0.267)
I/σ(I)	12.39 (1.77)	12.94 (1.99)	13.32 (1.77)	10.95 (1.50)	9.33 (0.97)	14.00 (2.07)
CC 1/2	0.999 (0.545)	0.999 (0.698)	0.999 (0.517)	0.998 (0.667)	0.998 (0.624)	0.997 (0.607)
Completeness (%)	99.96 (99.93)	99.85 (99.98)	99.91 (99.96)	99.95 (99.98)	99.88 (99.88)	99.95 (99.85)
Redundancy	10.5 (10.8)	10.6 (9.9)	10.6 (10.8)	10.6 (10.5)	10.6 (10.0)	10.7 (10.9)
**Refinement**						
R_{work}/ R_{free}	0.1896 (0.3020) 0.1983 (0.3241)	0.2070 (0.4891) 0.2343 (0.5092)	0.1916 (0.3651) 0.2128 (0.3573)	0.1756 (0.2991) 0.1971 (0.2929)	0.2018 (0.3739) 0.2240 (0.3912)	0.1815 (0.3267) 0.2070 (0.3394)
No. non-hydrogen atoms	2880	2781	2889	2853	2782	2870
Protein	2569	2560	2550	2547	2536	2548
Ligand/Ion	53	56	55	65	53	65
Water	258	165	284	241	193	257
B-factors (Å^2)	45.30	60.97	49.39	47.37	49.10	47.23
Protein	44.65	61.06	49.03	46.84	48.93	46.43
Ligand/Ion	74.84	79.46	68.82	67.48	61.41	68.21
Water	45.77	53.44	48.85	47.52	48.01	49.86
R.m.s deviations						
Bond lengths (Å)	0.006	0.009	0.013	0.018	0.010	0.016
Bond angles (°)	0.84	1.09	1.11	1.28	1.09	1.23
Ramachandran (%)						
favored	96.45	97.42	97.10	97.74	97.10	97.10
allowed	3.55	2.58	2.90	2.26	2.90	2.90
outliers	0	0	0	0	0	0

*Values in parentheses are for highest-resolution shell.

Table S3: List of the extended and *in silico* screened compounds. Naming based on here used nomenclature and PDB ligand codes. Added phenolic fragments are highlighted following the color coding in Figure 6.

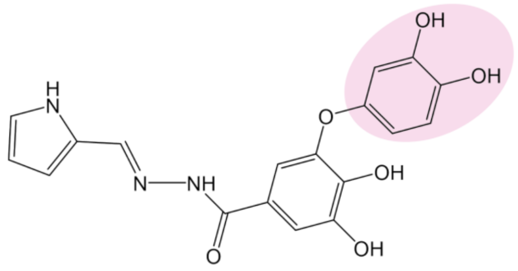	**H1 + HE9**
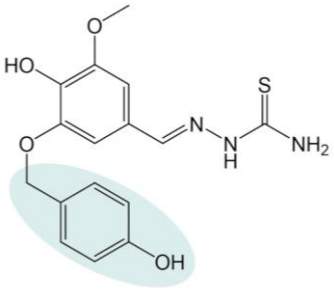	**T1 + HBA**
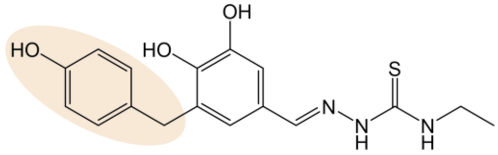	**T2 + YRL**
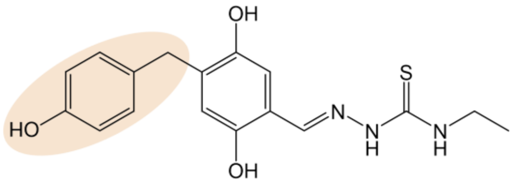	**T3 + YRL**
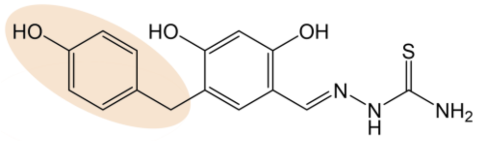	**T4 + YRL**
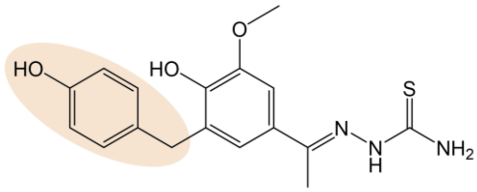	**T5 + YRL**

Synthesis of compounds (Supplementary Information)

All reagents of commercial quality were used without further purification. The purity of the synthesized compounds was determined by elemental analysis and verified to be ≥ 95%, by using a FlashSmart CHNS analyzer (Thermo Fisher) with gas-chromatographic separation. ^{1}H-NMR spectra were recorded at 25 °C on a Bruker Advance 400 MHz FT spectrometer. The ATR-IR spectra were recorded by means of a Spectrum Two (Perkin Elmer) spectrophotometer by using a diamond crystal plate in the range of 4000-400 cm^{-1}. Electrospray mass spectral analyses (ESI–MS) were performed with an electrospray ionization (ESI) time-of-flight Micromass 4LCZ spectrometer. MS spectra were acquired in positive EI mode by means of a DEP-probe (Direct Exposure Probe) mounting on the tip a Re-filament with a DSQII Thermo Fisher apparatus, equipped with a single quadrupole analyzer.

The thiosemicarbazones T1-T5 and the hydrazone H1 were prepared following reported procedures (Rogolino et al., 2015, 2017; Carcelli et al., 2016). Briefly, to a solution of the aldehyde in absolute ethanol, an equimolar amount of thiosemicarbazide was added, dissolved in the same solvent. The mixture was refluxed for 4-6 h, cooled at room temperature and concentrated in vacuum. The resulting precipitate was filtered off, washed with cold ethanol and dried in vacuum.

N-(2-pyrrolidyl)-3,4,5-trihydroxybenzoylhydrazone (H1). White powder. Yield = 25%. ^{1}H-NMR (DMSO-d6, 25°C), δ: 6.12 (s, 1H; ArH), 6.43 (s, 1H, ArH), 6.89 (s, 3H, ArH), 8.24 (s, 1H; HC=N), 8.75, 9.10 (s, br, 3H; OH), 11.20 (s, br, 1H; NH), 11.44 (s, br, 1H; OH). MS (EI, 70 eV), m/z (%) = 261.0 ([M]+ , 15); IR (cm^{-1}): ν(NH+OH) = 3215 (br); ν(C=O) = 1600. Anal. Calcd. for $C_{12}H_{11}N_3O_4$·: C 55.17; H 4.24; N 16.09. Found: C 55.22; H 4.43; N 15.79.

N-(3,5-dimethoxy-4-hydroxybenzyliden)-thiosemicarbazone (T1). Brown solid. Yield: 90%. ^{1}H-NMR (DMSO-d6, 25 °C), δ: 11.32 (s, 1 H, NNH); 8.80 (s, 1 H, OH); 8.13, 7.99 (s + s, 1 H + 1 H, NH_2); 7.92 (s, 1 H, CH = N); 7.05 (s, 1 H, ArH); 3.81 (s, 6 H, OCH_3). EI-MS: m/z = 255.0 [M + H]$^{+}$. Anal. Calcd. for $C_{10}H_{13}N_3O_2S$: C 50.19, H 5.48, N 17.56, S 13.40. Found: C 50.41, H 5.67, N 17.59, S 13.71.

N-(3,4-dihydroxybenzylidene)-thiosemicarbazone (T2). White powder. Yield = 72 %. ^{1}H-NMR (DMSO-d6, 25 °C), δ: 1.15 (t, 3H, CH_3), 3.58 (m, 2H, CH_2), 6.76 (d, 2H; ArH), 7.00 (d, 1H, ArH), 7.20 (s, 1H, ArH), 7.89 (s, 1H, HC=N), 8.29 (tr, br, 1H; NH), 8.96 (s, br, 1H, OH), 9.44 (s, br, 1H, OH), 11.15 (s, 1H; NH). MS (EI, 70 eV), m/z (%) = 239 ([M]$^{+}$, 100); IR (cm^{-1}): ν(NH) = 3381, 3293; ν(OH) = 3144 (br); ν(C=N) = 1625; ν(C=S) = 1545, 935. Anal. Calcd. for $C_{10}H_{13}N_3O_2S$: C 50.19; H 5.48; N 17.56; S 13.40. Found: C 50.45, H 5.39, N 14.20; S 13.45

N-(2,5-dihydroxybenzylidene)-thiosemicarbazone (T3). White powder. Yield = 51 %. ^{1}H-NMR (DMSO-d6, 25 °C), δ: 1.14 (t, 3H, CH_3), 3.57 (m, 2H, CH_2), 6.68 (s, 2H; ArH), 7.27 (s, 1H; ArH), 8.30 (s, 1H, HC=N), 8.40 (tr, br, 1H; NH), 8.82 (s, 1H, OH), 9.20 (s, 1H; OH), 11.30 (s, 1H, NH). MS (EI, 70 eV), m/z (%) = 239 ([M]$^{+}$, 100); IR (cm^{-1}): ν(NH) = 3385; ν(OH) = 3153 (br); ν(C=N) = 1547; ν(C=S) = 1533, 972. Anal. Calcd. for $C_{10}H_{13}N_3O_2S$: C 50.19; H 5.48; N 17.56; S 13.40. Found: C 50.28, H 5.76, N 17.69; S 13.54

N-(2,4-dihydroxybenzylidene)-thiosemicarbazone (T4). Yield = 78 %. ^{1}H-NMR (DMSO-d6, 25 °C), δ: 6.25– 6.29 (m, 2H; ArH), 7.67 (d, 1H, J = 8.4 Hz, ArH), 7.74 (s, br, 1H, NH), 7.94 (s, br, 1H, NH), 8.24 (s, 1H; HC=N), 9.78 (s, br, 2H, OH), 11.17 (s, br, 1H; NH). MS (EI, 70 eV), m/z (%) = 211.0 ([M]$^{+}$, 100); IR (cm^{-1}): νNH = 3479, 3347; νOH = 3262, 3128 (br); νC=N = 1630; νC=S =

1582, 875. Anal. Calcd. for $C_8H_9N_3O_2S$ 1/3H_2O: C 44.23; H 4.48; N 19.34. Found: C 43.94, H 4.05, N 19.18.

N-(3-methoxy-4-hydroxy-acetophenone)-thiosemicarbazone (T5). Yellow solid. Yield: 87%. ^{1}H-NMR (DMSO-d_6, 25 °C), δ: 10.03 (s, 1 H, NNH); 9.34 (s, 1 H, OH); 8.22, 7.87 (2 s, 1 H + 1 H, NH_2); 7.49 (d, 1 H, J = 2 Hz, CH_{Ar}); 7.26 (dd, 1 H, J = 8 Hz, J' = 2 Hz, CH_{Ar}); 6.76 (d, 1 H, J = 8 Hz, CH_{Ar}); 3.84 (s, 3 H, OCH_3); 2.25 (s, 3 H, CH_3). EI-MS: m/z = 240.0 $[M + H]^+$.

References

Bacchi, A., Carcelli, M., Compari, C., Fisicaro, E., Pala, N., Rispoli, G., et al. (2011). Investigating the role of metal chelation in HIV-1 integrase strand transfer inhibitors. *J Med Chem* 54, 8407–8420. doi:10.1021/jm200851g.

Carcelli, M., Rogolino, D., Bartoli, J., Pala, N., Compari, C., Ronda, N., et al. (2020). Hydroxyphenyl thiosemicarbazones as inhibitors of mushroom tyrosinase and antibrowning agents. *Food Chem* 303, 125310. doi:10.1016/j.foodchem.2019.125310.

Carcelli, M., Rogolino, D., Gatti, A., De Luca, L., Sechi, M., Kumar, G., et al. (2016). N-acylhydrazone inhibitors of influenza virus PA endonuclease with versatile metal binding modes. *Sci Rep* 6, 31500. doi:10.1038/srep31500.

Chen, L.-R., Wang, Y.-C., Lin, Y. W., Chou, S.-Y., Chen, S.-F., Liu, L. T., et al. (2005). Synthesis and evaluation of isatin derivatives as effective SARS coronavirus 3CL protease inhibitors. *Bioorg Med Chem Lett* 15, 3058–3062. doi:10.1016/j.bmcl.2005.04.027.

Rogolino, D., Bacchi, A., De Luca, L., Rispoli, G., Sechi, M., Stevaert, A., et al. (2015). Investigation of the salicylaldehyde thiosemicarbazone scaffold for inhibition of influenza virus PA endonuclease. *J Biol Inorg Chem* 20, 1109–1121. doi:10.1007/s00775-015-1292-0.

Rogolino, D., Gatti, A., Carcelli, M., Pelosi, G., Bisceglie, F., Restivo, F. M., et al. (2017). Thiosemicarbazone scaffold for the design of antifungal and antiaflatoxigenic agents: evaluation of ligands and related copper complexes. *Sci Rep* 7, 11214. doi:10.1038/s41598-017-11716-w.
